# Dual-initiation promoters with intertwined canonical and TCT/TOP transcription start sites diversify transcript processing

**DOI:** 10.1038/s41467-019-13687-0

**Published:** 2020-01-10

**Authors:** Chirag Nepal, Yavor Hadzhiev, Piotr Balwierz, Estefanía Tarifeño-Saldivia, Ryan Cardenas, Joseph W. Wragg, Ana-Maria Suzuki, Piero Carninci, Bernard Peers, Boris Lenhard, Jesper B. Andersen, Ferenc Müller

**Affiliations:** 1 Biotech Research and Innovation Centre, Department of Health and Medical Sciences, University of Copenhagen, Ole Maaloes Vej 5, 2200 Copenhagen, Denmark; 2 Institute of Cancer and Genomic Sciences, College of Medical and Dental Sciences, University of Birmingham, Edgbaston, Birmingham B15 2TT UK; 3 Institute of Clinical Sciences, Faculty of Medicine, Imperial College London, W12 0NN London, UK; 4 MRC Clinical Sciences Centre, Hammersmith Hospital Campus, W12 0NN London, UK; 5 Laboratory for Molecular Biology and Genetic Engineering, GIGA-R, Université de Liège, Liège, Belgium; 6 Department of Biochemistry and Molecular Biology, Faculty of Biological Sciences, University of Concepcion, Concepción, Chile; 7 Division of Genomic Technologies, RIKEN Center for Life Science Technologies, 1-7-22 Suehiro-cho, Tsurumi-ku, Yokohama, 230-0045 Japan; 8 Laboratory for Transcriptome Technology, RIKEN Center for Integrative Medical Sciences, Yokohama, Kanagawa 230-0045 Japan

**Keywords:** Developmental biology, Gene regulation, Non-coding RNAs, Transcription, Transcriptional regulatory elements

## Abstract

Variations in transcription start site (TSS) selection reflect diversity of preinitiation complexes and can impact on post-transcriptional RNA fates. Most metazoan polymerase II-transcribed genes carry canonical initiation with pyrimidine/purine (YR) dinucleotide, while translation machinery-associated genes carry polypyrimidine initiator (5’-TOP or TCT). By addressing the developmental regulation of TSS selection in zebrafish we uncovered a class of dual-initiation promoters in thousands of genes, including snoRNA host genes. 5’-TOP/TCT initiation is intertwined with canonical initiation and used divergently in hundreds of dual-initiation promoters during maternal to zygotic transition. Dual-initiation in snoRNA host genes selectively generates host and snoRNA with often different spatio-temporal expression. Dual-initiation promoters are pervasive in human and fruit fly, reflecting evolutionary conservation. We propose that dual-initiation on shared promoters represents a composite promoter architecture, which can function both coordinately and divergently to diversify RNAs.

## Abstract (web summary)

The functional significance of start site choice in promoter architectures is little understood. Here the authors identify in zebrafish development and mammalian cells a class of dual-initiation promoters, in which non-canonical YC dinucleotides reflecting 5’-TOP/TCT initiation are intertwined with canonical YR-initiation.

## Introduction

Transcription is a tightly regulated process initiated by RNA polymerase II (Pol II) in the core promoter region, which is typically −40 to +40 nucleotides with respect to transcription start sites (TSS). There are no universal core promoter elements^1^ as they are diverse in their sequence and functions, and the structure–function relationship of core promoters remains poorly understood. Sequencing of capped RNA 5′ ends by CAGE (cap-analysis of gene expression) revealed that an overwhelming majority of TSSs are anchored by a purine base at the start site (+1 position) and flanked by pyrimidine in the upstream region (−1 position), thus defining consensus Y_−1_R_+1_ (hereafter called YR-initiation) as canonical initiator in mammals^2^ and in teleosts (zebrafish and tetraodon)^3^, suggesting generality of conserved initiator among vertebrates. Analysis of core promoters in *Drosophila melanogaster* revealed a related but more motif-like TC_−1_A_+1_GT initiator sequence^4,5^. In contrast, transcription initiation of translation-associated genes (ribosomal proteins, snoRNA host genes, translation initiation, and elongation factors) is anchored by C_+1_ (cytosine) and flanked by a polypyrimidine stretch^6–11^. These non-canonical initiators have previously been termed 5′-TOP (terminal oligo-polypyrimidine) in mammalian systems or TCT initiators in *Drosophila*^12^ (hereafter called YC-initiation) and these YC-initiation-dependent genes were shown to be conserved in zebrafish^3^. *Drosophila* ribosomal protein genes with TCT promoters are recognized by a TFIID-independent transcription initiation mechanism and bound by the TATA-binding protein (TBP) family member TBP-related factor 2 (TRF2)^13^. These results suggest that the non-canonical initiation is specialized for a subset of genes and facilitates a non-canonical initiation complex formation with distinct proteins from that of TBP and TFIID, likely reflecting distinct regulation of transcription initiation^14^. While other, rare non-canonical initiation types exist, such as TGTT^15^ and GAA_(+1)_G initiation^3^, however, these have not yet been supported by independent biochemical validation, therefore we focus our study on YC-initiations. It is unknown, why such a non-canonical initiation has evolved and been maintained in evolutionary distant species. Important insight into potential functional significance of the non-canonical initiation is emerging from studies investigating target genes of mTOR pathways that are translationally regulated^16,17^, and enriched in 5′-TOP/TCT initiator. The 5′-TOP initiator is defined by a minimum of 4–15 pyrimidine sequences^18^. The polypyrimidine stretch proximal to the 5′ end of these genes is a target for translation regulation and has been suggested to serve as a target mechanism for oxidative and metabolic stress, or cancer-induced differential translational regulation by the mTOR pathway^16,17,19–21^. The existence of 5′-TOP/TCT promoters raises the questions of how widespread non-canonical initiation is and the nature of its relationship with canonical initiation.

We have previously generated CAGE datasets^3^ in zebrafish and profiled all transcription initiators during embryogenesis from the maternal to zygotic transition (MZT) and then through organogenesis. We performed a comprehensive and unbiased analysis of TSSs in promoters and characterized the features and roles of non-canonical initiation by a systematic survey of the base composition within the TSSs in CAGE datasets^3^. This analysis led us to uncover non-canonical YC-initiation in thousands of genes that are proximal to or intertwined with the canonical YR-initiation in the same core promoter region, thus revealing thousands of what we term dual-initiation (DI) promoter genes. We provide multiple lines of evidence for the functional relevance of dual-initiation. Our genome-wide analyses of initiation usage in development has uncovered differential usage of initiators, differential response of initiators during translation inhibition and selective association of snoRNA biogenesis, which is predicted to be processed by splicing from introns of the YC-initiation products of dual-initiation genes. We thus demonstrate that the two initiation types within promoters represent a composite of promoter architectures and reflect two regulatory functions, which can generate distinct sets of RNAs with different post-transcriptional fates. Our findings highlight another level of complexity of core promoter regulation during development, and broaden the scope for functional dissection of overlaid promoter architectures that act in the complexity of the developing embryo.

## Results

### Non-canonical YC-initiation

**Fig. 1:**
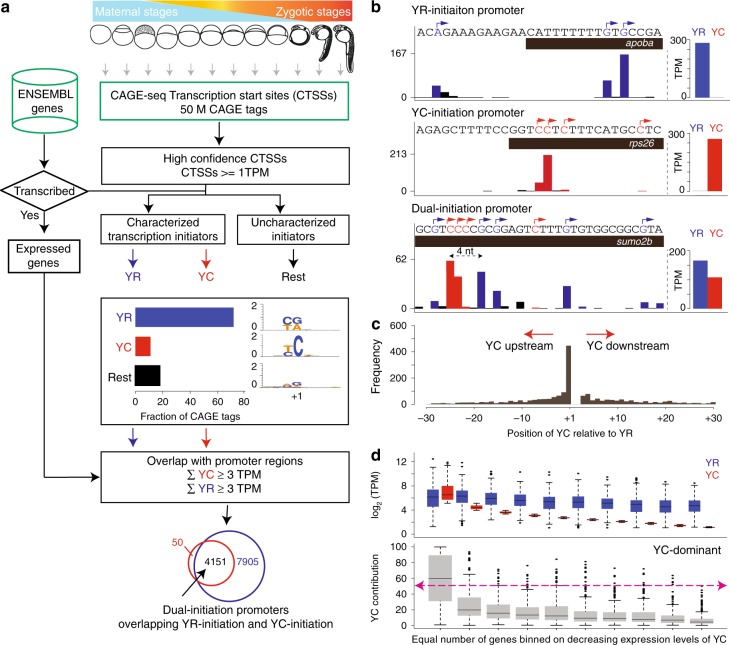
Intertwined canonical initiator (YR) and non-canonical initiator YC (alias as TCT/5’TOP) within the same core promoter. **a** A systematic pipeline for identification of canonical (YR) and non-canonical (YC) initiators in the zebrafish developmental promoterome. CTSSs are classified into YR and YC initiators based on CAGE transcription start sites (CTSSs). **b** UCSC browser views with CAGE data from prim 5 stage to illustrate examples of YR-initiation (*apoba*) and YC-initiation (*rps26*) promoters along with a gene promoter with intertwined YR-initiations and YC-initiations (*sumo2b*). YR-initiations and YC-initiations are shown in blue and red colors respectively. Barplot on the right shows the sum of expression levels of YR-initiations and YC-initiations. Highest CTSS represents the dominant transcription start site. The distance between dominant YR and YC in *sumo2b* is four nucleotides. **c** Frequency of position of dominant YC-initiation relative to dominant YR-initiation in dual-initiation promoters. **d** Contribution of YC-initiation with respect to YR-initiation expression levels in prim 5 stage. The 4151 genes with dual-initiation are sorted according to YC expression levels and grouped into 10% bins. Expression levels (top) and proportion of YC-initiation to total (bottom) are shown. Red dash line indicates 50% cutoff. TPM, tags per million.

To comprehensively map non-canonical initiation events at single nucleotide resolution, we have reanalyzed published CAGE data of RNA start base distribution by pooling CAGE Transcription Start Sites (CTSSs) with at least 1 tag per million (TPM) across 12 stages in zebrafish embryo development^3^ (Fig. 1a). The majority of CTSSs (71.6%) have canonical (Y_−1_R_+1_) start sites (Fig. 1a; Supplementary Fig. 1a). Importantly, a substantial proportion of TSSs possess a non-canonical pyrimidine initiation (labeled Y_−1_C_+1_ in Fig. 1a, Supplementary Fig. 1a). The remaining CTSSs include RNAs with a well-characterized GG dinucleotide associated with post-transcriptional processing products^3^, Drosha-processing sites on pre-miRNAs^22^, snoRNA 5′-end capping events^23^ and other uncharacterized non-canonical start base events, unlikely to reflect true transcription start. These were excluded from further analysis. The majority of YR-initiation (85.97%) and YC-initiation (83.05%) sites mapped within the expected promoter region of ENSEMBL transcripts (500 bases upstream and 300 bases downstream) and thus support detection of true transcription initiation products. YR-initiation and YC-initiation are highly reproducible across replicates (Supplementary Fig. 1b). For downstream analysis, we retained only those robustly detected transcripts that are transcribed in at least two developmental stages and whose promoter expression level is at least 3 TPM. At this filtering threshold, 4201 promoters have YC-initiation and 12,056 promoters have YR-initiation (Supplementary Data 1). Intersection analysis of gene promoters revealed that 50 (1.2%) genes carry only YC-initiation and 7905 (65.5%) genes have only YR-initiation, thus regulated by a single type of initiator. However, the majority of YC-initiation site-containing promoters (98.8%) also carry YR-initiation sites (Fig. 1a). We have termed this class of promoters as dual-initiation (DI) promoters (Fig. 1b). DI promoters identified by CAGE were also confirmed by independently generated nAnTi-CAGE^24^ data from seven developmental stages with a high degree of overlap (Supplementary Fig. 1c, d; Supplementary Data 2). We further validated dual-initiation promoters by capped mRNA sequencing at prim 5 stage of development (24 h post fertilization), which, though less sensitive than CAGE, has demonstrated high frequency of dual-initiation events and demonstrated statistically significant overlap with CAGE and nAnTi CAGE detected dual-initiation promoter genes at prim 5 stage (Supplementary Fig. 1e). The lower efficiency of capped RNA-seq in detecting DI promoters is attributed to its lower sensitivity in detecting lower levels of YC-initiation (Supplementary Fig. 1f), disproportionately affects detection of the YC component of DI promoters.

For all dual-initiation promoter genes, we summed the expression levels of all YR and YC components and genes were classified as either YR-dominant or YC-dominant depending upon the expression levels of their YR and YC components. The *sumo2b* gene (Fig. 1b) has a higher total level of YR-initiations than YC-initiation, thus classified as a YR-dominant gene. We then used the highest expression level of YR and YC CTSSs and determined the position of dominantly used YR and YC TSS. The YR-dominant TSS is located 4 nucleotides downstream of the YC-dominant TSS in the *sumo2b* gene (Fig. 1b). The distance between dominant YR-initiation and YC-initiation of all DI promoters at prim 5 stage fall mostly within 30 bases, with a notable spike in usage of YC directly upstream to YR CTSS position (Fig. 1c). The enrichment for YC-initiation immediately upstream to YR CTSS (Fig. 1c) was detected in 18.1% (*n* = 512) of 2826 DI promoters at prim 5 stage and was independently verified in capped RNA-seq data (Supplementary Fig. 1g). The overall close proximity between the two types of initiations suggest that the initiation machineries, involved in controlling transcription of these transcripts recognize the same core promoter region. Comparing the expression levels of YR and YC components revealed that the contribution of YC-initiation to the total activity of dual-initiation promoters tends to be relatively small (Fig. 1d; Supplementary Fig. 1g), resulting in only a small portion (8.3%; *n* = 251) of genes as YC-dominant in prim 5 stage (Fig. 1d). However, YC-initiation can be dominant over YR-initiation in individual genes, even at lowly expressed promoters (Fig. 1d; Supplementary Fig. 1h). In conclusion, we show that non-canonical YC-initiation events are pervasively intertwined with canonical YR-initiation and occur within a small physical distance within the same core promoter regions.

### Features of dual-initiation gene promoters

**Fig. 2:**
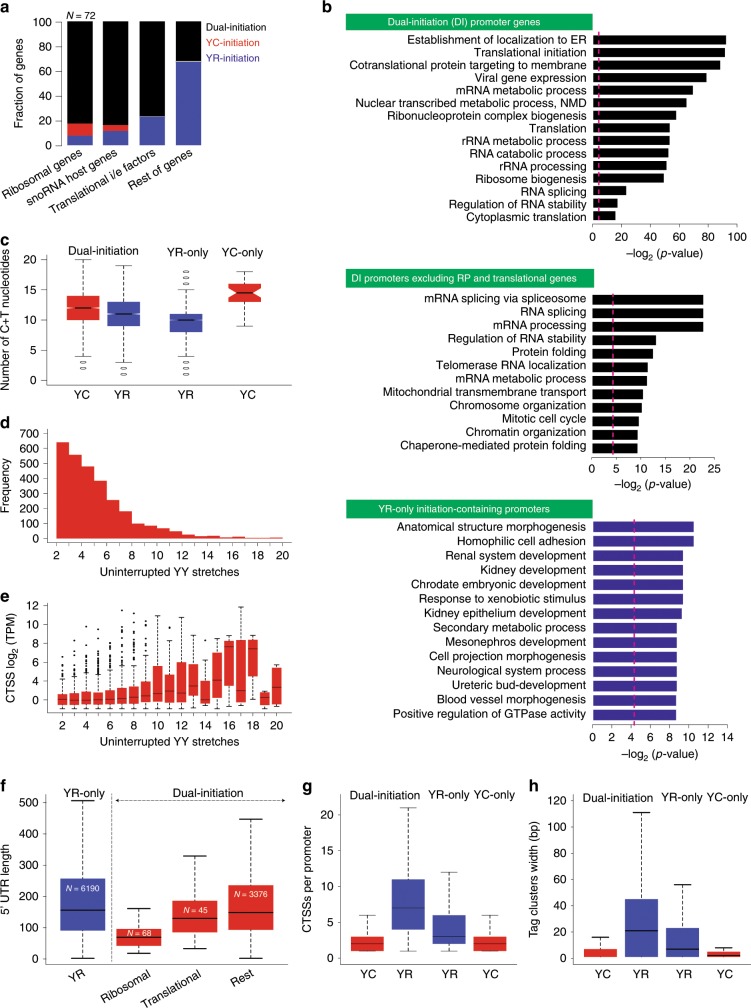
Characteristic features of dual-initiation and single initiation promoter genes. **a** Stack bar of proportion of single/dual-initiation promoter genes among translation-associated gene families as indicated. **b** Gene ontology (GO) categories of single and dual-initiation promoter genes clustered as indicated in green fields. **c** Sequence composition around dominant YR-initiation and YC-initiation sites of single/dual-initiation promoters. **d**, **e** Presence of polypyrimidine stretches in DI promoters. *X*-axis indicates the length of uninterrupted pyrimidine stretch with respect to YC-initiation frequency (**d**) and expression levels of YC-initiation sorted by increasing frequency of uninterrupted polypyrimidine stretches (**e**). **f** 5′ UTR length of dual-initiation and single initiation YR genes. **g** Frequency of CTSS in single/dual-initiation promoter genes. **h** Tag cluster width of single/dual-initiation promoter genes. Boxplots show the 5th, 25th, 50th, 75th, and 95th percentiles where center line is the median.

Translational-associated genes such as ribosomal proteins, translation initiation/elongation factors and small nucleolar RNA (snoRNA) host genes are transcribed by 5′-TOP/TCT initiators, thus we asked whether their zebrafish homologs possess single or dual-initiation. The annotation of zebrafish snoRNAs is not comprehensive, therefore we analyzed a size selected RNA library^25^ enriched for full-length snoRNA length (18–250 nt) and annotated 176 novel zebrafish snoRNAs (Supplementary Data 3). Intersection of the expressed genes from the above listed gene-families revealed that most of these genes carry dual-initiation sites (Fig. 2a). Gene ontology (GO) analysis of DI promoter genes revealed an enrichment of translation machinery components (translation, translation elongation, and translation termination), co-translational proteins targeted to membrane,RNA stability and nonsense mediated decay (Fig. 2b; Supplementary Data 4). Enrichment of ribosome-related functions is consistent with previous studies describing YC-initiation^18,26^ associated with such genes, while our findings reveal a dual-initiation mechanism featuring these promoters (Fig. 2a). Excluding translation-associated genes from the query list revealed an enrichment of additional unexpected GO terms such as mRNA splicing via spliceosome, telomerase RNA localization, chromosome organization and mitotic cell cycle (Fig. 2b; Supplementary Data 4). In contrast, YR-only initiator genes are enriched for GO terms related to morphogenesis, pattern specification, and embryonic development (Fig. 2b) characteristic of the prim 5 stage of development and highlight the functional distinction of core promoter architectures.

Sequence composition around dominant TSSs of both initiation sites revealed a greater fraction of pyrimidines (C/T) in sequences adjacent to YC-initiation sites (Fig. 2c), predominantly with an uninterrupted stretch of at least 4 pyrimidines (Fig. 2d), a characteristic feature of the 5′-TOP motif (reviewed in the ref. ^18^). We find that the longer an uninterrupted pyrimidine stretch around YC-initiation, the higher the expression level of dominant YC CTSSs (Fig. 2e). Translation-associated genes carry a longer stretch of pyrimidines (Supplementary Fig. 2a), which is in agreement with the stringent definition of translationally regulated 5′-TOP mRNAs^16^. Dual-initiation promoter genes have shorter 5′-UTR length as compared to single initiation YR promoters (Fig. 2f), which may reflect efficient translation, as transcripts with longer 5′UTR tend to have lower translational efficiency^27^.

Next, we sought to define the promoter features of YR-components and YC-components of dual-initiation promoters. CAGE defined TSSs have revealed three main classes of promoter shapes, namely broad peak, sharp peak and bimodal peaks^2^. 5′-TOP/TCT promoters were primarily associated with sharp peak promoters of highly expressed genes^1^. To explore the promoter features of dual-initiation genes, we first calculated the number of CTSSs and observed that dual-initiation genes have a higher number of YR-initiation sites (an average of 6 CTSSs) as compared to their YC constituent (an average of 2 CTSSs) or YR-only genes (an average of 3 CTSSs) (Fig. 2g). Accordingly, the YR component of dual-initiation promoters are composed of wider tag clusters than those by YC-initiation (Fig. 2h). We then asked if positionally-constrained motifs characteristic of known promoter architectures can be assigned to either YC and YR-initiation events in DI promoters. We have plotted YR, YY, SS, WW (Y=C/T; R=A/G; S=C/G; W=A/T) dinucleotides and positionally constrained motifs (TATA box, GC box, and CCAT motif) with respect to YR and YC-initiation events at fertilized egg and at prim 5 stage. The WW dinucleotide (W-box motif) present in most promoters in zebrafish^28^, is enriched in both initiators in the fertilized egg, but depleted in prim 5 stage (Supplementary Fig. 2b, c). Frequency of CC and TC dinucleotides are comparable between single and dual-initiation promoters therefore, base frequency does not explain enrichment for YC usage in dual-initiation promoters (Supplementary Fig. 2d). The finding that YC-initiation is associated with positionally-constrained motifs of YR-initiation, suggests that YC-initiation can utilize previously described promoter regulation mechanisms. Moreover, we have detected similar developmental utilization of sequence determinants of YC transcription start site choice to that previously described for YR-initiation^28^. TATA, CCAT and GC box motifs, however, were not enriched with either initiation events in both stages (Supplementary Fig. 2b, c).

Taken together these observations support the suggestion that DI promoter is a promoter classification category encompassing a large number of promoters in the zebrafish genome. DI promoters represent a composite of canonical and 5′-TOP/TCT promoter features and are used not only by translation-associated genes but a wider range of GO categories.

### Differential regulation of YC and YR-initiation

**Fig. 3:**
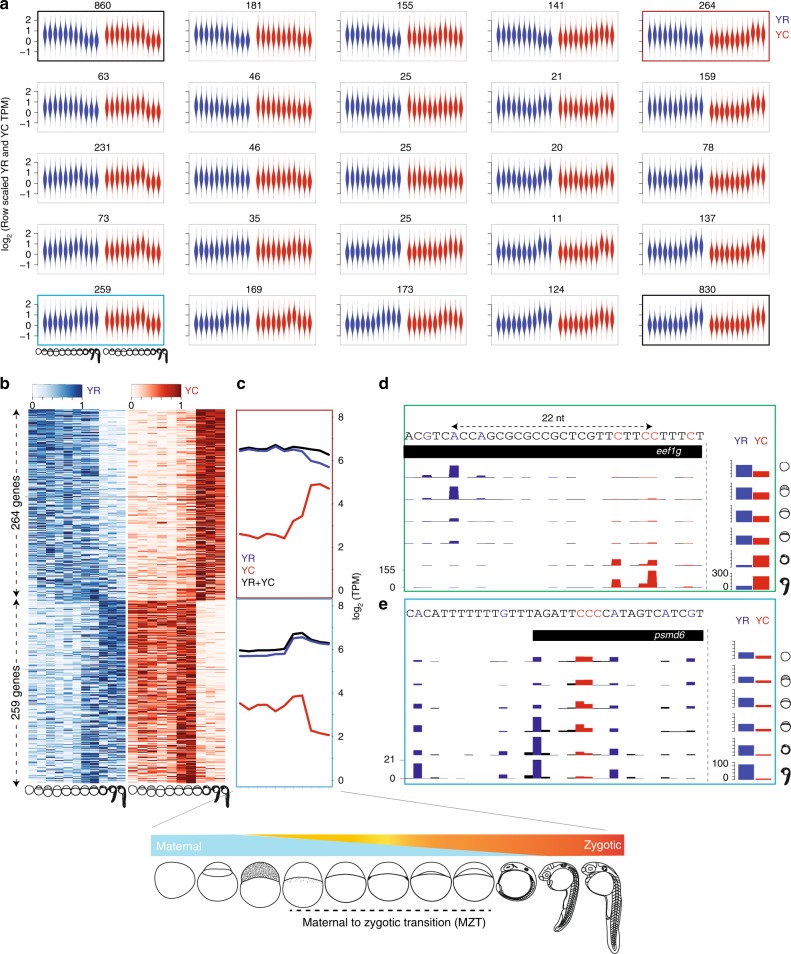
Maternal to zygotic transition of YR-initiation and YC-initiation. **a** Violin plot of expression profiles (tags per million) of YR and YC components of genes during embryo development clustered by Self Organizing Map (SOM) analysis. Blue and red colors indicate YR and YC components respectively. *X*-axis represents developmental stages as indicated. *Y*-axis indicates expression levels scaled between YC and YR components separately. Numbers indicate genes in the cluster. **b** Heatmap rows show the gene expression profiles with YR and YC-initiation of selected SOM clusters indicated by the gene numbers on the left. Expression values are scaled row-wise between 0 to 1, separately for YR and YC. Genes are ranked by statistical significance of anticorrelation. Black line indicates significance threshold (−0.5). **c** Averaged expression level of total (black) YR-initiation (blue) and YC-initiation (red) across the clustered group of genes shown in **b**. **d**, **e** UCSC genome browser views of CTSSs for the *eef1g* and *psmd6* gene promoters. Barplots on the right shows the sum of CTSSs of YR-initiation and YC-initiation events respectively. Distance between dominant YC and YR-initiation is indicated in **d**.

We have previously shown that two distinct and independently regulated promoter sequence codes, such as the W-box and +1 nucleosome positioning signals, are often overlaid in individual promoters and used differentially during the maternal to zygotic transition of embryo development^28^. The existence of such overlapping sequence codes, together with the observation that TCT promoters and canonical initiator may be regulated by different initiation complexes^12,13^ prompted us to hypothesize that intertwined YR-initiation and YC-initiation events may represent differential regulatory principles. Thus divergent regulatory inputs may target dual-initiation promoters and lead to divergent transcriptional regulation during embryo development. Therefore, we asked about the relationship between the expression dynamics of YR-initiation and YC-initiation during early embryo development. We performed self-organizing map (SOM) clustering between YR and YC expression levels for 4151 DI promoter genes and observed the typical zebrafish developmental expression profiles, characterized by combinations of two opposing trends. A typical maternal-dominant trend includes a relatively stable mRNA pool at early stages originating from the oocyte, which is removed by RNA degradation after zygotic genome activation and manifesting as dramatic reduction of maternal transcripts, typically after the 7th stage analysed in Fig. 3a (e.g., right panels of first row). An opposite zygotic dominant trend features low or no maternal activity followed by the zygotic activation, most pronounced after the 7th stage (e.g., Figure 3a bottom row panels). In most of the clusters YC and YR-initiated RNAs follow similar trends. However, several clusters are characterized by distinct profiles for YR and YC components, where the YR component is expressed predominantly maternally then reduced zygotically, whereas the YC component showing an opposite trend (Fig. 3a, red frame). Another opposing trend between YC and YR-initiation is also seen with YC being predominantly maternal with YR being primarily zygotic (Fig. 3a, blue frame). These trends are traceable in individual genes with YR and YC components, showing opposite maternal/zygotic dominance, indicating that they are distinctly subjected to maternal mRNA degradation and corresponding zygotic transcription activation^28–30^ (Fig. 3b, c; Supplementary Data 5). The opposing trends of YC and YR-initiation events are followed by individual CTSS within a promoter as demonstrated by genome browser views of the *psmd6* gene(Fig. 3e) and the *eef1g* translation elongation factor gene (Fig. 3d), the human homolog of which is transcribed by a non-canonical YC-type initiator^18^. These findings of independent regulation of YC and YR components of gene promoters was also verified in the same set of genes by an independent nAnTi-CAGE-seq experiment carried out at representative stages of maternal to zygotic transition (Supplementary Fig. 3a–c). These results indicate that YR-initiation and YC-initiation are not specific to but can be selectively used at either maternal or zygotic stages by individual genes, which suggests that YC-initiation and YR-initiation of genes can respond to differential regulatory inputs. Taken together, the expression dynamics within these subsets of dual-initiation promoters indicate independent regulation of YR-initiation and YC-initiation components, which is apparent during the dramatic overhaul of the transcriptome at the MZT.

### YC components regulate snoRNA expression

**Fig. 4:**
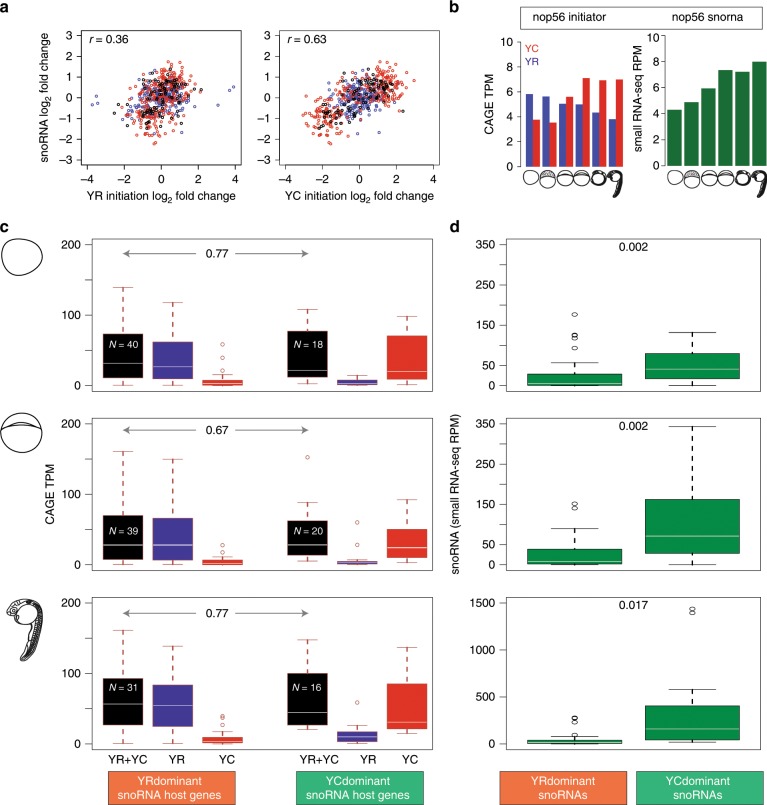
Correlation of expression levels of YR and YC components of snoRNA host genes with that of snoRNA expression levels. **a** Scatter plot of correlations between snoRNA log fold changes with YR-initiation log fold changes (left), and with YC-initiation log fold changes (right) across six developmental stages among 94 dual-initiation snoRNA host genes. Each point corresponds to one stage-gene pair of YR-dominant transcripts (blue), YC-dominant transcripts (red) and the rest (black). **b** Bar plot of CAGE expression levels (tpm) of YR (blue) and YC (red) components of initiation at the *nop56* gene. Expression levels of snoRNA (dark green) were calculated from small RNA-seq data in reads per million. **c** Box plot of TPM expression levels of YR-initiation (blue), YC-initiation (red), and total (black) of YR-dominant and YC-dominant snoRNA host genes selected for comparable total expression level are shown. The number of YR-dominant and YC-dominant snoRNA host genes are denoted by “*N*”. **d** Box plot of expression levels of corresponding snoRNAs (green) from YR-dominant and YC-dominant host genes shown in **d**. Developmental stages are fertilized egg, 30% epiboly and prim 5 stages (top to bottom) are depicted on the left. Boxplots show the 5th, 25th, 50th, 75th, and 95th percentiles where center line is the median.

snoRNAs are transcribed by host gene promoters, and are spliced out from introns of primary transcripts and subsequently form a riboprotein complex^31^. snoRNA host genes may carry two functional entities; snoRNA genes and their coding or non-coding host gene. Interestingly, a non-coding host gene (*GAS5)* of snoRNA^6^ was recently shown to have an additional function in maintaining nodal signaling^32^. In contrast to previous studies in mammals that described snoRNA host genes being transcribed by YC-initiation (5′-TOP/TCT), we showed that zebrafish snoRNA host genes carry dual-initiation (Fig. 2a). These observations raise the question, whether the dual function of snoRNA host genes is coupled to YR or YC-initiation and whether the two initiation events contribute selectively to snoRNA production. Indeed, it was previously shown that a 5′-TOP promoter element determines the specific ratio of snoRNA to mRNA production and an artificial canonical YR-initiation containing Pol II promoter is incompatible with the efficient release of snoRNA^11^. The dramatic dynamics of maternal and zygotic transcriptomes and the uncovered differential regulation of YC-initiation and YR-initiation at MZT, provides an opportunity to dissect differential regulation of snoRNA host gene products. We thus hypothesized that expression dynamics of YR and YC derived transcripts during MZT could be informative to trace the source RNA for embedded snoRNA genes in dual-initiation promoter host genes. To this end, first we plotted the correlation between the expression levels of both YR and YC components of 88 snoRNA host genes (containing 246 snoRNAs) and the expression of snoRNAs^25^ at the corresponding developmental stages (Fig. 4a). This analysis revealed a stronger correlation of the YC component (*r* = 0.63) with the expression of snoRNAs (Fig. 4a left and right panels), suggesting YC-initiation better explains snoRNA expression than YR-initiation. We have repeated these correlation analyses with an independent nAnTi-CAGE dataset at the developmental stages indicated on Fig. 4b and Methods section, and obtained similar results indicating YC-initiation (*r* = 0.61) correlating better than YR-initiation (*r* = 0.32) with snoRNA expression (Supplementary Fig. 4a).

To further explore transcription initiation patterns in snoRNA production we investigated the temporal dynamics of the *nop56* host gene, which shows increasing snoRNA expression between maternal to zygotic stages and corresponding increase in YC-initiation, but with a contrasted downward trending YR-initiation (Fig. 4b). We then globally analyzed snoRNAs expression levels in relation to the expression levels of YR and YC components of their host genes at three key stages during the maternal to zygotic transition. We have classified snoRNA host genes into YR-dominant and YC-dominant groups in each stage and plotted the expression levels of YR-components and YC-components of host promoter and the corresponding snoRNAs. This comparison of expression levels, indicates high snoRNA expression tending to cluster with high YC-initiation in all stages tested (Supplementary Fig. 4b). To quantitate these observations, we plotted the expression levels of total, YC and YR contribution of YC and YR-dominant host genes and compared them globally to snoRNA expression levels (Supplementary Fig. 4c, d). Total expression levels of YR and YC dominant genes vary greatly at different stages and do not allow distinguishing YC and YR-initiation contributions in this bulk analysis. Therefore, we further analyzed snoRNA expression in subsets of host genes with comparable expression levels, but with significantly varying contribution of YR and YC-initiation (YR and YC-dominant groups, Fig. 4c, d). In this comparison total expression levels are comparable (Fig. 4c), while snoRNAs expression levels are significantly higher in YC-dominant genes (Fig. 4d). The snoRNA expression differences between YC-dominant and YR-dominant genes follow YC expression changes in the corresponding CAGE data (compare Fig. 4c, d). Taken together, the various correlation analyses of divergent temporal expression patterns and levels of YR and YC-initiation suggests that YC-initiation better explains snoRNA expression than YR-initiation in dual-initiation promoters of host genes. Nevertheless, the localization of snoRNAs in many ribosomal and translation factors suggests that snoRNAs are produced together with the translation and rRNA biogenesis protein machinery encoded by their host genes and hence they are likely also co-regulated.

### Expression and localization of snoRNA and host RNA in embryos

**Fig. 5:**
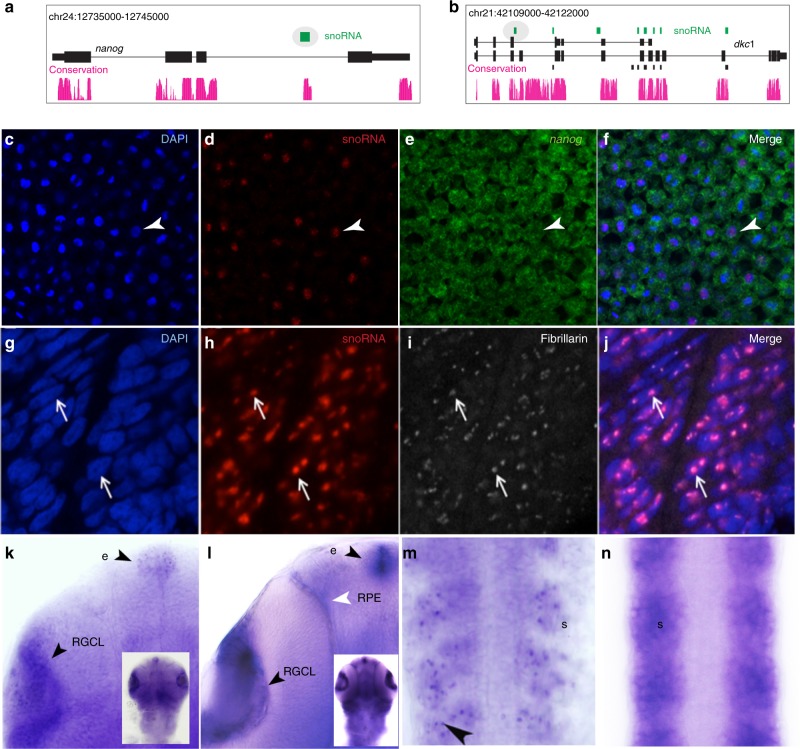
Localization of snoRNAs and host mRNA products in the embryo. **a**, **b** A UCSC browser showing annotated snoRNAs (green) in the introns of *nanog* and *dyskerin* (*dkc1*). Ensembl annotated genes and snoRNAs are shown as black tracks. Teleost sequence conservation tracks are shown in magenta. Two snoRNAs selected for expression analysis are highlighted in oval. **c**–**e** In situ hybridization in whole mount zebrafish embryos at the 30% epiboly stage with probes detecting nuclei (**c**), snoRNA gene embedded in *nanog* (**d**) *nanog* coding exon (**e**), and overlay (**f**). ARrowheads indicate overlapping spots. **g**–**j** In situ hybridization with snoRNA probe (**h**) for the *dyskerin* gene is detected in the nucleoli of somitic nuclei detected by DAPI (**g**), and indicated by immunohistochemical detection of fibrallin (**i** and overlay in **j**). Arrows indicate the same spots in the overlapping frames. **k**–**n** In situ hybridization probes detect *dyskerin* and embedded snoRNA gene activities in long-pec stage embryos. **k**, **m** snoRNA probe detects expression in epiphysis (arrowhead with **e**), retinal ganglion cell layer (arrowhead with RGLC) and the somites (arrowhead in **m**). **l**, **n** Exon probe for *dyskerin* indicates cytoplasmic expression in the epiphysis (arrowhead and **e**), retinal ganglion cell layer (arrowhead RGCL), and retinal-pigmented epithelium (white arrowhead and RPE) and the somites (s, black arrowhead in **n**). Inserts in **k** and **l** show dorsal views of heads, from which the magnified views are cropped.

The above results suggest that snoRNA host transcripts may be divergently expressed. However, their temporal expression dynamics may not reveal the full extent of differential RNA regulation that emerge from dual-initiation promoter genes. Therefore, we investigated the spatial expression patterns of two newly annotated snoRNAs (Supplementary Data 3) embedded in the intron of host gene *nanog* (Fig. 5a) and *dyskerin* (*dkc1*) (Fig. 5b), respectively. The snoRNA in *nanog* is conserved among teleosts (Fig. 5a) and is validated by RT-PCR (Supplementary Fig. 5a). The maternally expressed host gene *nanog* encodes a transcription factor, which regulates genome activation during early zebrafish development^30,33^ with no reported function in rRNA biogenesis. The *nanog* gene carries YR-dominant initiation and low level of mostly but not exclusively zygotic YC-initiation with corresponding low levels of snoRNA expression (Supplementary Fig. 5b, c). An antisense probe raised against the snoRNA was detected in some, but not all nuclei of zebrafish embryos at the sphere stage, whereas an exonic probe detects *nanog* distinctly in the cytoplasm in most cells, indicating the differential transcriptional and/or post-transcriptional fates of the two RNA products generated by the dual-initiation promoter (Fig. 5c–f).

In contrast to the *nanog* example, a snoRNA produced from the *dyskerin (dkc1)* gene (Fig. 5b) and validated by RT-PCR (Supplementary Fig. 5d) shows largely similar expression pattern with its host gene (Fig. 5k–n). This shared expression may be expected from their likely shared role in pseudouridylation of ribosomal RNA. The *dkc1* gene carries YR-dominant initiation in both maternal and zygotic stages (Supplementary Fig. 5e, f), while 3 of 4 minor YC-initiation sites become activated higher in zygotic stages (Supplementary Fig. 5e, f). Expression of the snoRNA by in situ hybridization in whole mount embryos revealed co-localization with Fibrillarin in highly expressing tissues, thus verifying the expected nucleolar expression profile (Fig. 5g–j). Expression of snoRNA in nucleoli were detected as speckles in nuclei of a subset of cells at long-pec stage, notably in the epiphysis, somatic muscle cells, and retinal ganglion cell layer (RGLC) of the eye. The host RNA *dkc1* exonic probe was detected ubiquitously in the cytoplasm with elevated activity largely in overlapping domains (e.g., epiphysis, RGLC and somites, with notable difference in *dkc1* signal in the retinal-pigmented epithelium (Fig. 5k–n). Taken together, these two examples demonstrate both differential subcellular localization and partially overlapping expression patterns of host gene products and their snoRNAs, consistent with potential divergence in both transcriptional and posttranscriptional regulation of these RNA products, generated from the same core promoter.

### Differential fates of YR and YC-initiation products

**Fig. 6:**
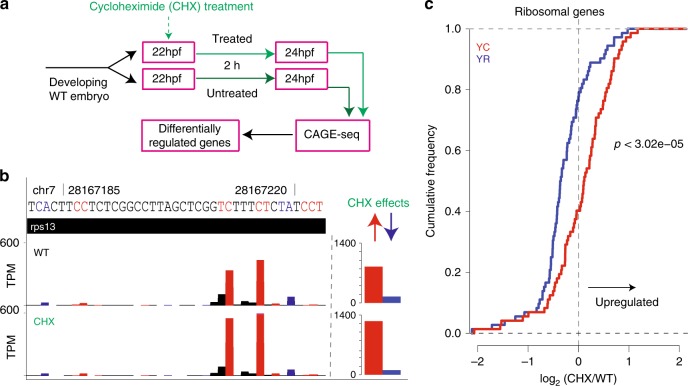
Differential regulation of YR-initiation and YC-initiation during translation inhibition. **a** Experimental design to study the response of YC-initiation and YR-initiation products during translation inhibition by cycloheximide. **b** A UCSC browser screenshot showing an example of levels of YR and YC components of the dual-initiation promoter gene *rps13*. The bar chart includes sum of all peaks. **c** Cumulative frequency of YR-initiation and YC-initiation of all ribosomal protein genes after cycloheximide treatment. *X*-axis indicates the log_2_ fold change of YR-initiation and YC-initiation in cycloheximide and wild type condition.

SnoRNA host genes are selectively subjected to nonsense mediated decay (NMD), shown by blocking NMD with the translation inhibitor cycloheximide, which led to stabilization of several (*UHG* and *GAS5)*^6,34^, but not all (e.g., *U17HG*^7^, *U87HG*^35^, *rpS16*^6^) snoRNA host genes. These results suggest differential stabilization of host RNAs due to differential association of snoRNA host mRNAs with translating ribosomes^7^. We asked whether dual-initiation promoter genes are potentially subjected to differential post-transcriptional/translational regulatory mechanisms involving NMD in zebrafish development. To test post-transcriptional regulation of YR and YC initiated RNAs, we blocked translation/NMD in zebrafish embryos by cycloheximide at 22 somites stage for 2 hours until prim 5 stage and performed CAGE analysis (Fig. 6a). We chose stages where YC-initiation is broadly active (Supplementary Fig. 1a; Fig. 3b), yet maternally deposited mRNAs have been cleared^36^. Thereby, post-transcriptional fates of de novo produced YC and YR-initiated products may be detected, excluding indirect effects of maternal mRNA stability. Overall, expression levels of zebrafish *gas5* mildly increased upon cycloheximide treatment with YC-initiation mildly upregulated and YR-initiation downregulated (Supplementary Fig. 6a), suggesting that *gas5* is regulated by NMD in zebrafish similarly to human, yet CAGE-based initiation profile analysis revealed differential fates of RNAs with YR-initiation and YC-initiation. To further demonstrate the response to cycloheximide by RNAs with distinct initiation sites within a single dual promoter, we highlight ribosomal protein gene *rps13* with multiple YR-initiations and YC-initiations (Fig. 6b). Expression levels of both YC-initiation products are upregulated, while YR-initiation products are downregulated, indicating that the intertwined YR-initiation and YC-initiations are significantly regulated in opposing directions (Fisher-exact test; *P* = 1.27e−07).

We next analyzed all ribosomal protein genes and observed YR-initiation and YC-initiation are differentially regulated (Kolmogorov-Smirnov test; *P* = 3.5e−05) upon cycloheximide treatment (Fig. 6c). YC-initiation was upregulated in 59.7% and YR-initiation was downregulated in 77.8% (43 and 56 out of 72, respectively) of ribosomal genes. However, at the individual gene level, distinct regulation of YC and YR-initiation is statistically significant for six genes only (five upregulated and one downregulated, Fisher’s exact test: *P*-adjusted ≤0.05, Supplementary Data 6). Subsequently, we have analyzed the response to cycloheximide on YR-dominant (*n* = 1774) or YC-dominant (*n* = 241) genes and observed significantly different response to cycloheximide (Kolmogorov-Smirnov test; P = 4.44e−05) in YC-dominant genes but not in YR-dominant genes. However, on the individual gene level the number of significant genes were negligible (Supplementary Fig. 6b, Supplementary Data 6). Taken together, these results suggest that either the fate or transcription of RNAs with distinct initiation bases can be differentially regulated in the YC-dominant subset of DI promoter genes upon cycloheximide treatment.

### Dual-initiation promoter genes are conserved across metazoans

**Fig. 7:**
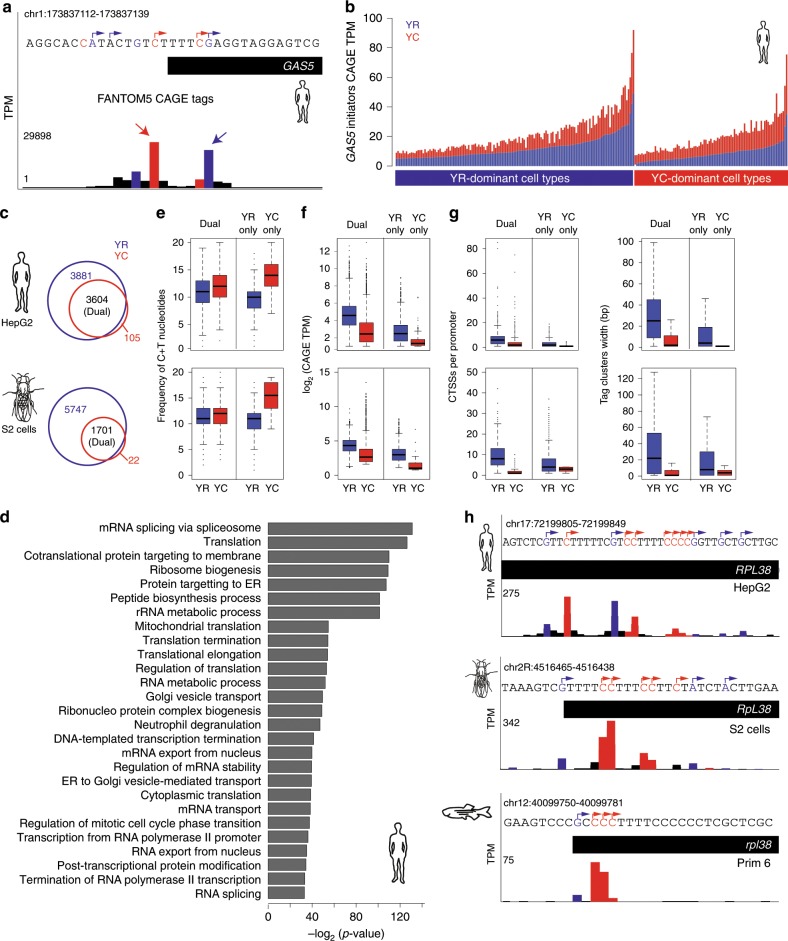
Dual-initiation (DI) promoters are conserved in human and *Drosophila*. **a** A UCSC browser screenshot of human *GAS5* promoter CAGE data (summed CTSSs) in FANTOM5 cell types. CTSSs show transcription of YR-initiation and YC-initiation within same core promoter region. **b** Expression levels of YR-initiations and YC-initiations by summing their CTSSs. Promoter are classified as YR-dominant or YC-dominant across individual cell types and their expression is shown in stacked bars. *Y*-axis shows the expression levels measured in tags per million (TPM). **c** Venn diagram with intersection of gene promoters with YR and YC-initiation in human HepG2 and *Drosophila* S2 cells. DI promoters are indicated in the overlap between detected YR-initiation and YC-initiation. **d** Enrichment of gene ontology terms of DI promoters in human HepG2 cell line. **e** Comparison of C+T sequence content around transcription start sites in DI promoters with YR-only or YC-only initiation promoter in human and drosophila. **f** Expression levels of DI promoter genes in human and *Drosophila*. **g** Frequency of CTSSs and promoter width of DI promoters in human and *Drosophila*. **h** UCSC browser screenshots showing CTSSs in the promoter region of *RPL38* gene in human, *Drosophila* and zebrafish. YR-initiation and YC-initiation peaks are colored as blue and red. Boxplots show the 5th, 25th, 50th, 75th, and 95th percentiles where center line is the median.

Finally, we asked whether DI promoters detected in zebrafish are present among other metazoans. We first re-analyzed transcription initiation of the human snoRNA host gene *GAS5*, which is transcribed by a 5′-TOP promoter^6^. Visual inspection of combined CTSSs from FANTOM5^23^ revealed that *GAS5* utilizes the expected YC-initiation as its dominant initiator (indicated by arrow) (Fig. 7a). There was, however, unexpected presence of YR-initiation at a comparable expression level. We measured the expression levels of both initiators in individual cell types across FANTOM5 libraries and observed unexpectedly higher levels of YR component of *GAS5* promoter activity than its YC component, in multiple cell types (Fig. 7b). This result demonstrates the presence and differential expression dynamics of two initiations in a dual-initiation promoter in mammals. We then analyzed DI promoters by adapting the pipeline described in Fig. 1a to CAGE-seq data in human HepG2 cell line^23^ and *Drosophila* S2 cells^37^, and in GRO-cap data from human K562 and GM12878 cell lines^38^. Among expressed genes, 3920 (45%) promoters in HepG2 and 1701 (16%) promoters in S2 cells have intertwined YR-initiation and YC-initiation within the same core promoter (Fig. 7c; Supplementary Data 7). We also predicted 3899 (42.0%) and 4362 (45.5%) DI promoters in human K562 and GM12878 cell lines from GRO-cap data (Supplementary Fig. 7; Supplementary Data 8). The YC-initiation is dominant in 11.8% and 8.0% of DI promoters in human HepG2 and *Drosophila* S2 cells, respectively (Supplementary Data 7). Furthermore, the intersection of human and zebrafish orthologous DI promoter genes revealed that 1171 (38.5%) genes share the DI promoter feature, indicating a high degree of conservation of DI promoters among vertebrates. Gene ontology analysis of DI promoter genes in human has revealed enrichment for translation regulation, mRNA stability, and RNA splicing in human (Fig. 7d), similar to that in zebrafish (Fig. 2b). This suggests, that what were previously classed as 5′-TOP/TCT promoters, are better described as DI promoters in several cell types, both in human and *Drosophila* and argues for redefining non-canonical initiator promoters in these metazoans.

We next sought to compare sequence content, by analysis of expression levels and promoter width of dual-initiation promoters, in human and *Drosophila*. In both species, DI promoters have higher C+T content around the TSS, as compared to YR-only promoters, but lower than YC-only promoters (Fig. 7e), similar to observations in zebrafish (Fig. 2c). Dual-initiation promoters are highly expressed compared to YR-only and YC-only initiation promoters, which appears to be a shared feature among all three species (Figs. 1d, 7f). Dual-initiation promoters have a higher number of CTSSs, resulting in broad promoter shapes, whereas the YC component forms narrow tag clusters, similar to zebrafish (Fig. 7g compare to Fig. 2g). The UCSC browser view of the orthologs of ribosomal protein gene *RPL38* show similar intertwining of YR and YC-initiation events among all three species (Fig. 7h). Taken together, these results demonstrate that DI promoters are pervasive and an evolutionary ancient phenomenon, with highly conserved promoter architecture and expression features shared among metazoans which together highlight the importance of this promoter structure organization in divergent animal systems.

## Discussion

**Fig. 8:**
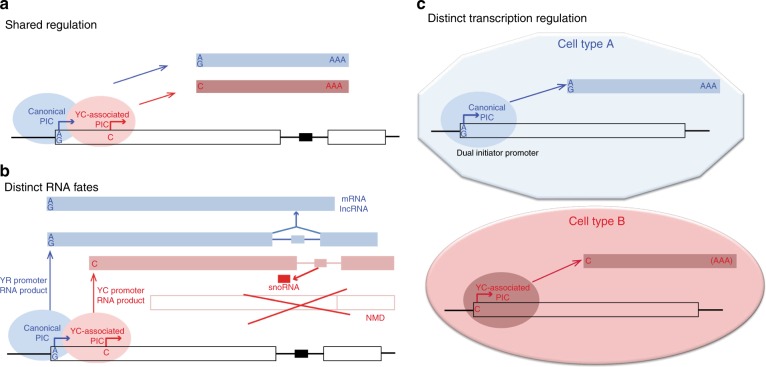
Models for utilization of dual-initiation promoters during development. **a** Dual-initiation promoters can be occupied by pre-initiation complexes (PIC) in a cell to generate two RNA products with distinct 5′ end base. These products can be produced on a shared promoter coordinately by partially overlapping PICs or by distinct PICs recruited coordinately. The co-regulation may occur on the cell or tissue level. **b** Model for differential utilization of YC and YR-initiation by divergent PICs to generate RNAs with distinct fate in the same or distinct cells. E.g. PICs form on the YR-initiation site for generating a protein coding mRNA or non-coding RNA gene product from a snoRNA host gene, while the YC-initiation may be utilized by a specialized PIC to produce an RNA which is processed to splice out snoRNAs and subjected to distinct degradation pathways. **c** A dual-initiation promoter is utilized divergently by YR and YC associated initiation complexes to adapt to requirements in different cells such as maternal activity in the oocyte versus zygotic activity in an embryonic cell.

In this study, we demonstrate the pervasive nature of non-canonical YC transcription initiation, intertwined with canonical YR-initiation, within the core promoter of thousands of genes in three model species. YC-initiation is utilized by a much larger set of genes than previously reported^6,7,12,18^. Recently, TCT initiation has been shown to be activated by a distinct set of core promoter binding transcription factors, highlighting its distinct function^39^ regulation by TBP family member TRF2^13^ and distinct enhancer interaction specificity^14^. Based on these features and the co-occurrence of the YC and YR-initiation sharing the same sequence platform, we propose that this dual-initiation arrangement represents a composite promoter architecture, which functions in coordinated as well as in divergently regulated forms. While we have found the two components of dual-initiation promoters mostly coregulated, we have also demonstrated their uncoupling during the maternal to zygotic transition (Fig. 8a). The independent regulation of initiation site selection in dual promoters during the MZT is not uniform among genes acting in the egg and the embryo, instead it appears to alternate among promoters by as yet unexplored rules. Nevertheless, the remarkable overlap of transcription initiation mechanisms on the same core promoter demonstrates how genes exploit core promoters to respond in more than one way to regulatory inputs in different ontogenic contexts (Fig. 8a).

We provide evidence that zebrafish snoRNA host genes are transcribed from YC-initiation, similar to other model systems^6,7^. However, we demonstrate that snoRNA host genes also carry canonical YR-initiation, not only in zebrafish, but in human cells. This opens the way for future investigation as to whether snoRNA host genes respond to distinct regulatory inputs to selectively direct host gene and snoRNA for distinct post-transcriptional fates. CAGE and RNA-seq by short read sequencing are not suitable to unequivocally uncouple the post-transcriptionally generated secondary RNA products from two initiation sites. Nevertheless, we show stronger association of YC-initiation than YR-initiation with snoRNA generation, by expression correlation analysis of initiation usage. Our results are in agreement with a previous study, which has demonstrated, that experimentally replacing YC-initiation (5′-TOP) in a snoRNA promoter with a YR-initiation site reduces snoRNA production^11^. Taken together, our observations strongly argue for a combination of transcription initiation mechanisms acting on snoRNA host genes and raises the question, whether the mixed nature of canonical and non-canonical initiators reflect a shared promoter region being used by two transcription initiation complexes. Such dual role of a promoter in a single ontogenic stage, potentially within the same cell, expands the transcript repertoire of that cell (see model in Fig. 8b) and could substantially impact on the as yet unexplored additional layer of diversity of RNAs produced from genes. Single cell CAGE technologies will be required in the future to verify co-regulation of the two initiation mechanisms in the same cell. We hypothesize, that the expansion of utilization of a non-canonical initiation to a wide range of genes could indicate a general transcription regulation paradigm, which represents adaptation to differential regulation of a variety of promoters^16,19^. Dual-initiation promoter genes are highly expressed compared to other genes (Figs. 1d, 7f). This is not purely due to the contributing transcription from YC components, as expression levels of the corresponding YR component alone is also higher than that of YR-only or YC-only initiator genes. This observation either suggest that sharing two alternative initiation mechanisms leads to a boost of expression levels, or suggest that YC-initiation might be evolutionary co-opted in highly expressed genes. It is interesting to note, that the efficiency of transcription correlates positively with translation efficiency and raises the possibility that highly expressed DI promoters contribute to coordination between transcription and translation^40^.

Important insight into potential functional significance of the non-canonical initiation comes from studies on target genes of the mTOR pathway that are translationally regulated^16,17^, and are enriched in 5′-TOP/TCT initiator. Polypyrimidine proximal to 5′ end of these genes is a target for translation regulation and may serve as a target in oxidative and metabolic stress, or cancer induced differential translation regulation by the mTOR pathway^16,17,19–21,41^. Other studies argue for the co-transcriptional regulation of post-transcriptional fates of RNAs, where promoter identity influences cellular localization and translation efficiency of mRNAs under different environmental conditions^42,43^. Thus, it is plausible that specialization of transcription initiation has co-evolved with post-transcriptional regulation to regulate RNA fates by transcription. Dual-initiation promoters offer the potential for linking translational regulation to transcriptional regulation in a large range of genes and thus increase the repertoire of genes that may respond to such signals. In this study we have identified many genes, which carry a low level of YC-initiation events, which may reflect a non-induced, ground state for YC regulation. However, there was a notable correlation between the length of polypyrimidine stretch at the 5′ end and the expression level of YC (Fig. 2e). It is not yet possible to distinguish in the CAGE dataset whether this correlation reflects RNA stability or transcriptional differences. Nevertheless, an unanswered question remains, whether the polypyrimidine stretch at the 5′-end is required for selective translation factor binding such as eIF4F complex, or also represent distinct transcription regulatory signals acting at the transcription initiation level.

The current definition of 5′-TOP mRNA includes a stretch of minimally 4–13 pyrimidines^18^, based on observations restricted to translational-associated genes^18^, which also have longer pyrimidine stretches in zebrafish (Supplementary Fig. 2d). This definition has been suggested to be potentially too stringent, as translationally regulated genes revealed by ribosome profiling are enriched in transcription initiation with “C” and carry only a short pyrimidine stretch^16,17^. We used a threshold of 1 TPM and identified thousands of YC-initiation sites and thus expanded the pool of genes that ought to be considered when transcriptomic responses to metabolic stress, for example via the mTOR pathway, are sought and our results argue for the need for the discrimination of RNAs produced from the same promoter, by using transcriptome analyses with single nucleotide resolution. Taken together, our findings provide a framework for future studies to understand coordinated regulation of transcription and translation of thousands of genes.

The unexpected widespread presence of YR and YC-initiation intertwined in the same core promoter raises a question as to why this pervasiveness was not seen before. Previous studies analyzing TSSs in a genome-wide level, reported multiple TSSs in same core promoter^2,3,5,23,28^, but downstream analyses were focused on dominant TSSs, the majority of which are YR, and as a result YC-initiation remained unexplored. Reinvestigation of human and *Drosophila* cell line datasets in this study demonstrated that the dual-initiation is a widespread phenomenon and share similar sequence features, promoter shapes, expression levels and enriched gene ontology. Thus, dual-initiation promoter genes in three major metazoan model systems suggest an evolutionary ancient, shared promoter architecture with fundamental multicellular function in development and motivates future investigation into the regulation and consequences of selective transcription initiation within gene promoters in general.

## Methods

### Zebrafish maintenance

All zebrafish strains were maintained in designated facility (according to UK Home Office regulations) in a recirculating system (ZebTEC, Tecniplast) at 26 °C in a 10-h dark, 14-h light photoperiod and fed three times daily.

Zebrafish experiments were restricted to early developmental stages and adults were only used for natural breeding. Animal work presented in this study was carried out under the project licenses 40/3681 and P51AB7F76 assigned to the University of Birmingham, UK.

### Zebrafish CAGE data after cycloheximide treatment

We generated zebrafish CAGE data for translation inhibition experiment. Zebrafish embryos were treated with 100 µg/ml cycloheximide (Sigma-Aldrich) or 0.1% DMSO as control for 2 hours, starting at 22 hours post-fertilization (hpf). Total RNA was extracted from the control and treatment groups at 24 hpf using TRIzol (Invitrogen/ThermoFisher) following the manufacturer’s instructions and used for CAGE libraries preparation as described before^3^, except for the use of oligo-dT primer instead of random-primers in the first strand synthesis step. CAGE libraries were sequenced on Illumina Genome Analyzer IIx system.

### No-amplification non-tagging CAGE (nAnTi-CAGE) sequencing

Total RNA was extracted from multiple stages of zebrafish development (Fertilized egg, 128 cell, 512 cell, 30% epiboly, 4 somite, Prim 5, and high pec using the miRNeasy kit (Qiagen), according to the manufacturer’s instructions. nAnT-iCAGE libraries were prepared as described in the ref. ^24^ using the CAGE™ Preparation Kit (DNAFORM). All libraries were sequenced on Illumina HiSeq2500 except the high pec library which has been sequenced on NextSeq500. Reads were trimmed to 27 bp and mapped to zebrafish Zv9 reference genome with bowtie1^44^, in default n-mode(maximum two mismatches in the 27-bp seed region), reporting only uniquely mapped reads (i.e., with -m 1 option. CTSS were called using CAGEr package^45^.

### RNA sequencing of capped RNAs

Total RNA was extracted from 24 hpf embryos using TRIzol reagent (ThermoFisher) and DNAse treated using TURBO DNA-free™ Kit (ThermoFisher) according to the manufacturer’s instructions. Full length cDNA libraries were prepared using TeloPrime Full-Length cDNA Amplification Kit (LexoGen), designed to capture 5′ Capped, polyadenylated transcripts. Two full cDNA libraries were prepared (technical replicates) according to the provided user manual, using 2 µg of total RNA as input, with differing numbers of PCR amplification cycles: 14 and 16, respectively. Sequencing libraries were prepared from both cDNA libraries using the MicroPlex-Library-Prep-Kit-v2 (Diagenode) and sequenced (2 × 100 bp reads) on HiSeq 2500 System (Illumina). For identification of transcription start sites, only reads starting with the 5′ TeloPrime adapter were selected, trimmed to 27 bp using cutadapt^46^ and mapped to the zebrafish Zv9 reference genome with Bowtie1^44^, in default n-mode(maximum two mismatches in the 27-bp seed region), reporting only uniquely mapped reads (i.e., with -m 1 option) CAGE-like TSS (CTSS) were called using CAGEr package^45^. In order to allow normalized values (tpm) to be comparable across all datasets capped RNAseq and the nAnTi CAGE data were normalized with the CAGEr package to fit a powerlaw distribution with slope 1.22 in the range between 10 and 1000 tag (read) counts, similar to the published CAGE data set in the ref. ^3^.

### Publicly available CAGE and GRO-cap data

CAGE data on zebrafish, human and *Drosophila* were downloaded from previous studies. Mapped zebrafish CAGE data was used from previous study^3^. Mapped human CAGE data was downloaded from FANTOM5^23^. Three replicates of HepG2 CAGE data was merged and converted CAGE tags count into tags per million (TPM). *Drosophila* CAGE raw reads was downloaded from modENCODE^37^. CAGE libraries were mapped using bowtie2^47^. We allowed two mismatches and only unique mapping reads were retained. Mapped reads having a “G” mismatch in the first nucleotide was corrected and transcription start site was adjusted accordingly. GRO-cap data from human K562 and GM12878 cell lines were downloaded from GEO database^38^. GRO-cap data were mapped using bowtie2^47^. We allowed two mismatches and only unique mapping reads were retained for downstream analysis.

### Downstream analysis of CAGE data

Based on −1 and +1 nucleotides for each CAGE Transcription Start Site (CTSS) we classified Y_−1_R_+1_ (Y: pyrimidine (C/T)) and (R: Purine (A/G)) as canonical initiator^2,3^ and Y_−1_C_+1_ as non-canonical initiator. For all analysis, we selected CTSS with a minimum expression level of 1 TPM in one of the 12 developmental stages. From the above pool of selected CTSSs, we intersected remaining CTSSs and included those CTSS with a minimum of 0.5 TPM. Canonical and non-canonical initiators were separately clustered if they overlapped within 20 nucleotides in the same strand resulting a tag clusters (TCs). Expression levels of all CTSS falling within the tag clusters are summed to give the expression level of tag clusters. CTSS with the highest expression level within the tag cluster, defines the dominantly used transcription start sites. The width of a tag clusters defines the promoter shape, which is classified as either sharp or broad. Gene expression levels are calculated by aggregating tag clusters in the assigned promoter regions (500 nucleotides upstream and 300 nucleotides downstream of Ensembl annotated TSSs). Expression level of canonical initiation of each gene were calculated by aggregating canonical CTSS. The expression level of non-canonical initiation of each gene were calculated by aggregating non-canonical CTSS. To determine whether a gene has dominant canonical initiation (referred to as YR-dominant) or dominant non-canonical initiation (referred to as YC dominant), we compared the sum of canonical and non-canonical initiation. When gene expression of canonical initiation is higher (>50%) than non-canonical initiation, the gene is defined as YR-dominant. Similarly, when the expression level of non-canonical initiation is higher than canonical initiation, gene is termed as YC dominant.

### Annotation of zebrafish snoRNAs

Size selected (18–350 nucleotide) zebrafish small RNA-seq data from six developmental stages (egg, high, 30% epiboly, 12 somites, prim 5, and prim 16) was downloaded from public dataset^25^. Adapters were filtered, and mapped sequence reads to zebrafish genome (zv9) using bowtie2^47^. Sequence reads were first mapped to ribosomal RNAs (rRNAs) and excluded those mapping to rRNAs. Unmapped reads were then remapped to genome by allowing up to four multimappings reads. To ensure that snoRNAs are annotated from mapped reads that resemble the expected full-length of snoRNAs, we retained only those mapped reads that longer than 50 nucleotides and potentially represent full-length snoRNAs rather than small RNA fragments. SnoRNAs were annotated by using four different tools, namely Infernal^48^, snoReport^49^, snoGPS^50^, and snoscan^51^. Infernal was used together with covariance model from RFAM^52^. An *e*-value cutoff of 0.05 for each covariate model provided by RFAM was used. SnoReport, snoscan, and snoGPS were used with default parameters for annotation of novel snoRNAs. To retain high confidence snoRNAs, we excluded snoRNAs that have low reads (<5 reads), residing on exons and repeats. Ensembl (version-79) has 312 annotated snoRNAs^53^ and 270 of them are supported by at least 5 reads in developmental stages we analyzed. Out of 270 snoRNAs from Ensembl, we predicted 264 snoRNAs and annotated 176 novel snoRNAs. We finally quantified snoRNAs expression by counting mapped reads using BEDTools^54^. Total mapped reads were calculated using SAMtools^55^ and then converted into reads per million.

### Gene ontology

Gene Ontology analysis was done by using GOstats package^56^ from BioConductor^57^. Over-represented GO terms were corrected for multiple testing with the Benjamini-Hochberg false discovery rate and obtained statistically significant GO terms by applying a *P*-value cutoff of ≤0.05.

### Data visualization

A genome browser view of multiple genes was downloaded from UCSC genome browser^58^. CTSSs and other relevant data were uploaded on UCSC Genome Browser as tracks for visualization. A screenshot of promoter regions with data tracks were downloaded from the UCSC browser. All other figures were made using R.

### RNA extraction and RT-PCR amplification

Purification of total RNA was performed using miRNeasy mini kit (Qiagen, Cat. 217004) following the manufacturer’s instructions. cDNA was synthesized using the iScript cDNA synthesis kit (BioRad, Cat. 170–8890) from 200 ng of purified RNA and snoRNA sequences were amplified by RT-PCR. Amplified cDNAs were verified by electrophoresis in 4% MetaPhor agarose gel (Lonza, Cat. 50184). We used the following primers for amplification: *dkc1*-snorna: TGATGAACTTGTTTATCCATTCGC and TGTCAGTCATGTATAATCATCTTGGC; nanog-snorna: CGTGTCCATGCTGTTGCTTG and CTTGTATCATCGTGCCTTTAAGACG.

### Fluorescent whole-mount in situ hybridization

T3 promoter was linked at the 5′ and the 3′ end of the full-length cDNA for each amplified snoRNAs for the synthesis of antisense and Sense riboprobes, respectively. Transcription were done by T3 polymerase using digoxigenin (DIG) labeling mix (Roche) or DNP-11-UTP (TSA™ Plus system, Perkin Elmer) according to manufacturer’s instructions. The probes were subsequently purified on NucAway spin columns (Ambion), and then ethanol-precipitated. Single whole-mount in situ hybridizations were performed as described previously^59^. Double fluorescent in situ hybridizations were carried out as described previously^60^.

### Whole mount immunofluorescence after ISH hybridization

Embryos were washed in wash buffer (0.3% PBS, v/v triton), incubated in blocking buffer (1× PBS, 0.1% tween, 4% goat serum, 1% BSA, 1% DMSO) for 3 h and then incubated with primary antibody overnight at 4 °C (Anti-Fibrillarin, Abcam 38F3, 1:10). Embryos were then washed in wash buffer and blocked 3 h followed by incubation with the secondary antibody overnight at 4 °C (Anti-Mouse Alexa 633, 1:500).

### Imaging

Microscopy images were obtained with an Olympus DP70 camera fixed on a BX60 Olympus microscope. Confocal imaging was performed using a Leica TCS SP5 inverted confocal laser microscope (Leica Microsystems, Germany) Digitized images were acquired using a 63× glycerol-immersion objective at 1024 × 1024 pixel resolution. Series of optical sections were carried out to analyse the spatial distribution of fluorescence, and for each embryo, they were recorded with a *Z*-step ranging between 1 and 2 μm. Image processing, including background subtraction, was performed with Leica software (version 2.5). Captured images were exported as TIFF and further processed using Adobe Photoshop and Illustrator CS2 for figure mounting.

### Reporting summary

Further information on research design is available in the Nature Research Reporting Summary linked to this article.

## Supplementary information

Supplementary Information

Description of Additional Supplementary Files

Supplementary Data 1

Supplementary Data 2

Supplementary Data 3

Supplementary Data 4

Supplementary Data 5

Supplementary Data 6

Supplementary Data 7

Supplementary Data 8

Supplementary Data 9

Supplementary Data 10

Supplementary Data 11

Supplementary Data 12

Supplementary Data 13

Reporting Summary

## Data Availability

The data that support this study are available from the corresponding author(s) upon reasonable request. Raw sequencing data for CAGE-seq and capped RNA-seq are publicly available at NCBI Sequence Read Archive under accession numbers SRA055273 and PRJNA575342. Processed and normalized CAGE-seq CTSSs, capped RNA-seq CTSSs and small RNA-seq data used in all analyses in this study are provided as Supplementary Data files (Supplementary Data 9–13).

## Code availability

CAGE data was analyzed using Bioconductor package CAGEr. All custom code is available upon request.
