# Supplementary material for: Dual-initiation promoters with intertwined canonical and TCT/TOP transcription start sites diversify transcript processing
Source: Nat Commun. 2020 Jan 10;11:168. doi: 10.1038/s41467-019-13687-0 (PMC6954239; doi:10.1038/s41467-019-13687-0)
Supplement: Supplementary file 1 — Supplementary Information [file 41467_2019_13687_MOESM1_ESM.pdf]

Supplementary Information for

Dual-initiation promoters with intertwined canonical and TCT/TOP transcription start sites diversify transcript processing

Nepal et al.

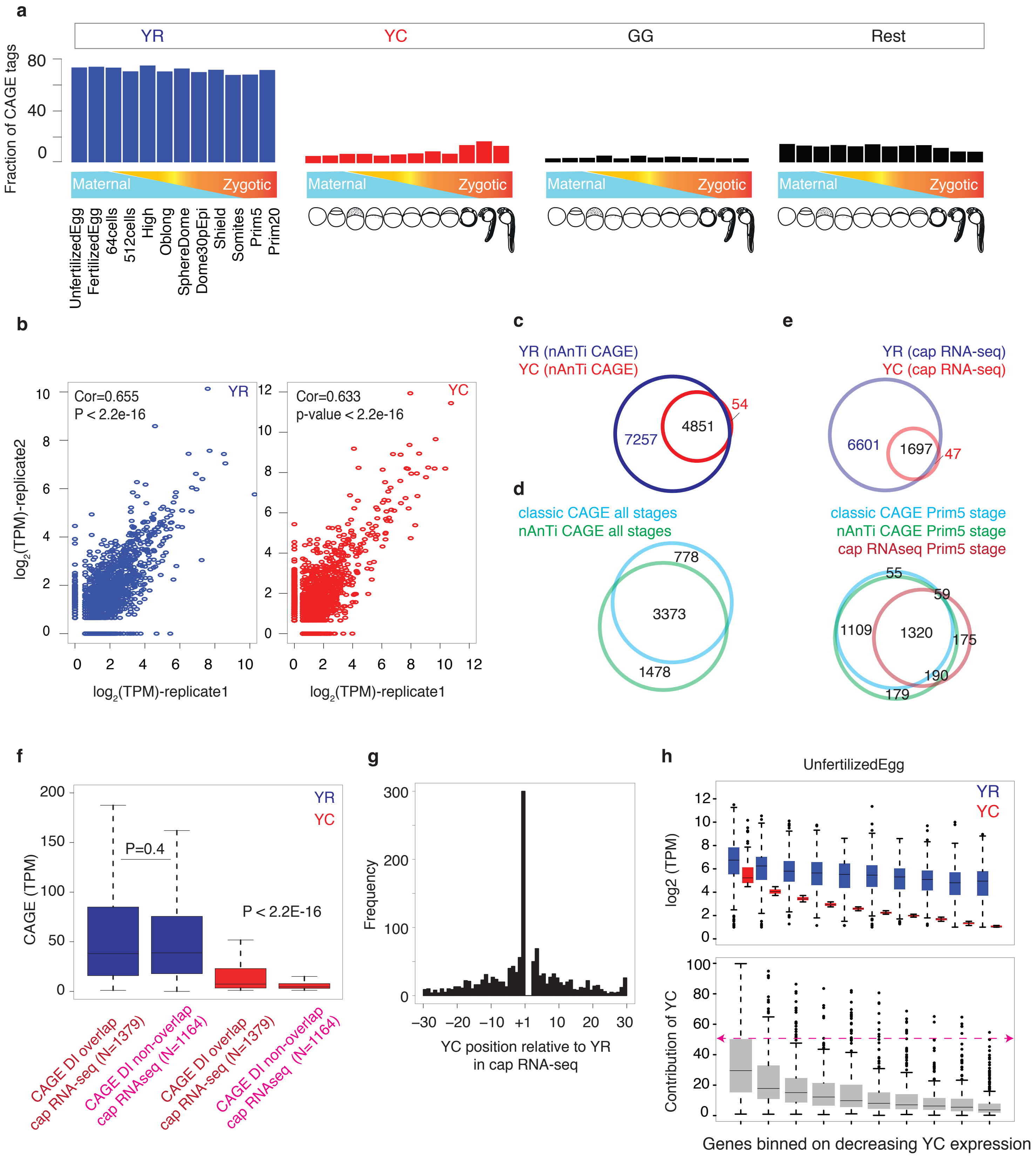

Supplementary Figure 1. Distribution and correlation of YC-initiaions and YR-initiations in zebrafish developmental transcriptomes. (**a**) Classification of CTSSs based on dinucleotide frequencies around CTSSs Y-axis indicates fraction of CTSSs. (**b**) Correlation of canonical YR-initiation and non-canonical YC-initiation between two replicates of prim 5 stage. (**c**) Venn diagram intersection of genes with YR-initiation and YC-initiation from nAnTi CAGE across 7 developmental stages. (**d**) Venn diagram intersection of dual-initiation genes from classic CAGE-seq and nAnTi CAGE-seq. (**e**) Venn diagram intersection of genes with YR-initiation and YC-initiation from cap RNA-seq data from prim 5 stage (top panel). Venn diagram intersection of dual-initiation promoters from classic CAGE-seq, nAnTi CAGE-seq and cap RNA-seq data from prim5 stage (bottom panel). (**f**) Comparison of expression level of YR and YC components of dual initiation genes from classic CAGE-seq (at prim 5) that are either supported (overlap) or unsupported (non-overlap) by cap RNA-seq data. (**g**) Frequency of YC-initiation relative to the position of the dominant YR-initiation in dual initiation promoters in capped RNA-seq data from prim 5 stage. (**h**) Box plot diagram of the contribution of YC and YR initiation to expression levels (top) and proportion of YC initiation (bottom) in the unfertilized egg stage. Genes are sorted according to YC expression levels and grouped into 10% bins.

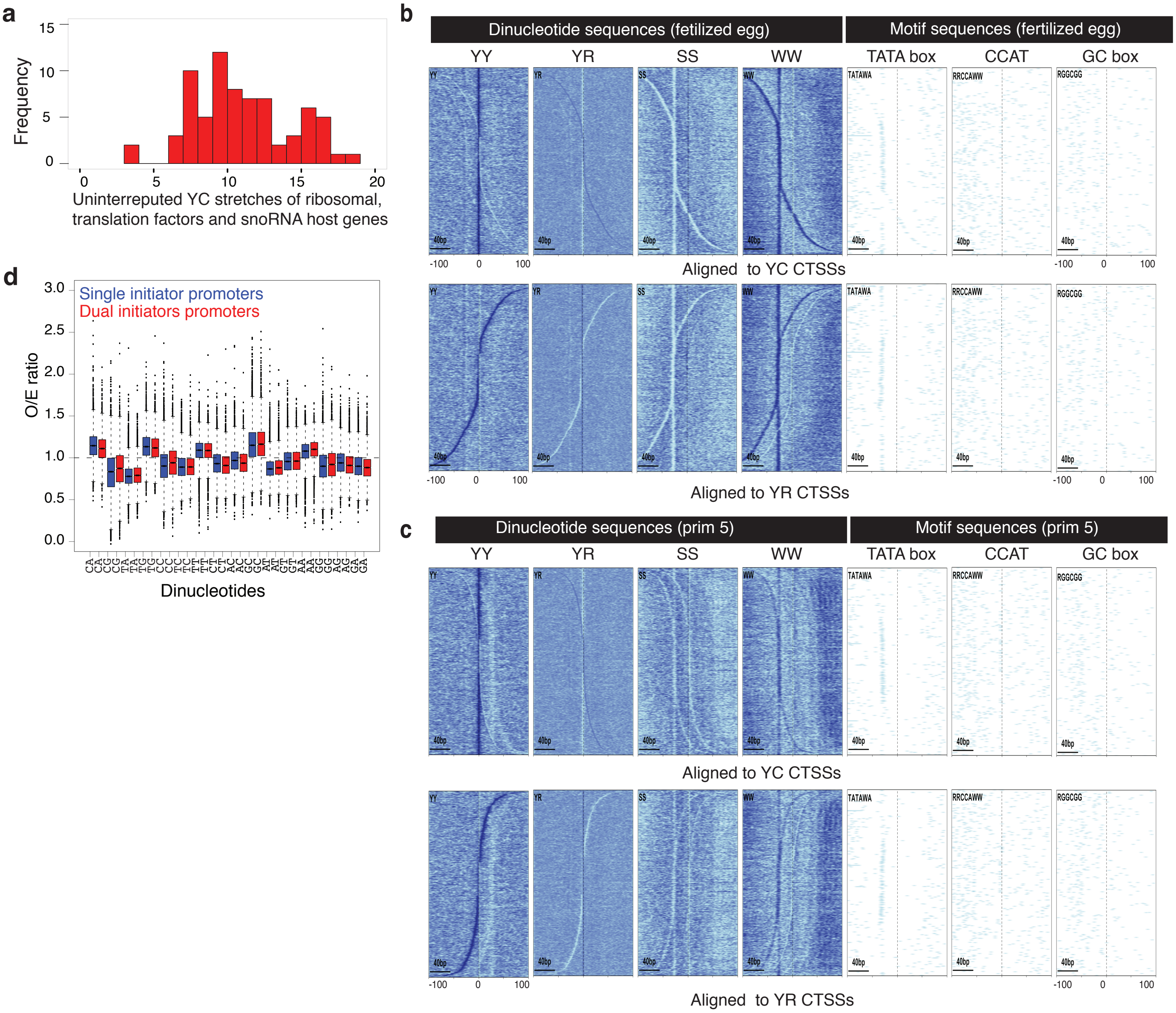

Supplementary Figure 2. Features of dual-initiation promoter genes. (**a**) Frequency of uninterrupted polypyrimidine stretches around YC-initiation sites of translational-associated genes (ribosomal proteins, translation initiation/elongation factors and snoRNA host genes). X axis indicate the maximum length of uninterrupted stretches of pyrimidine sequence. (**b-c**) Distribution of dinucleotide (YY/YR/SS/WW; Y=C/T; R=A/G; S=C/G; W=A/T) sequence content and (TATA, CCAT and GC box) motifs with respect to YR-initiation and YC-initiation of dual-initiation promoters in (**b**) fertilized egg and (**c**) prim 5 stage. Genes are aligned based on distance between YR and YC and aggregated to the +1 position of YR and YC dominant CTSS respectively. (**d**) Observed and expected ratio of dinucleotide sequences at the promoter region of dual initiation and single initiation genes.

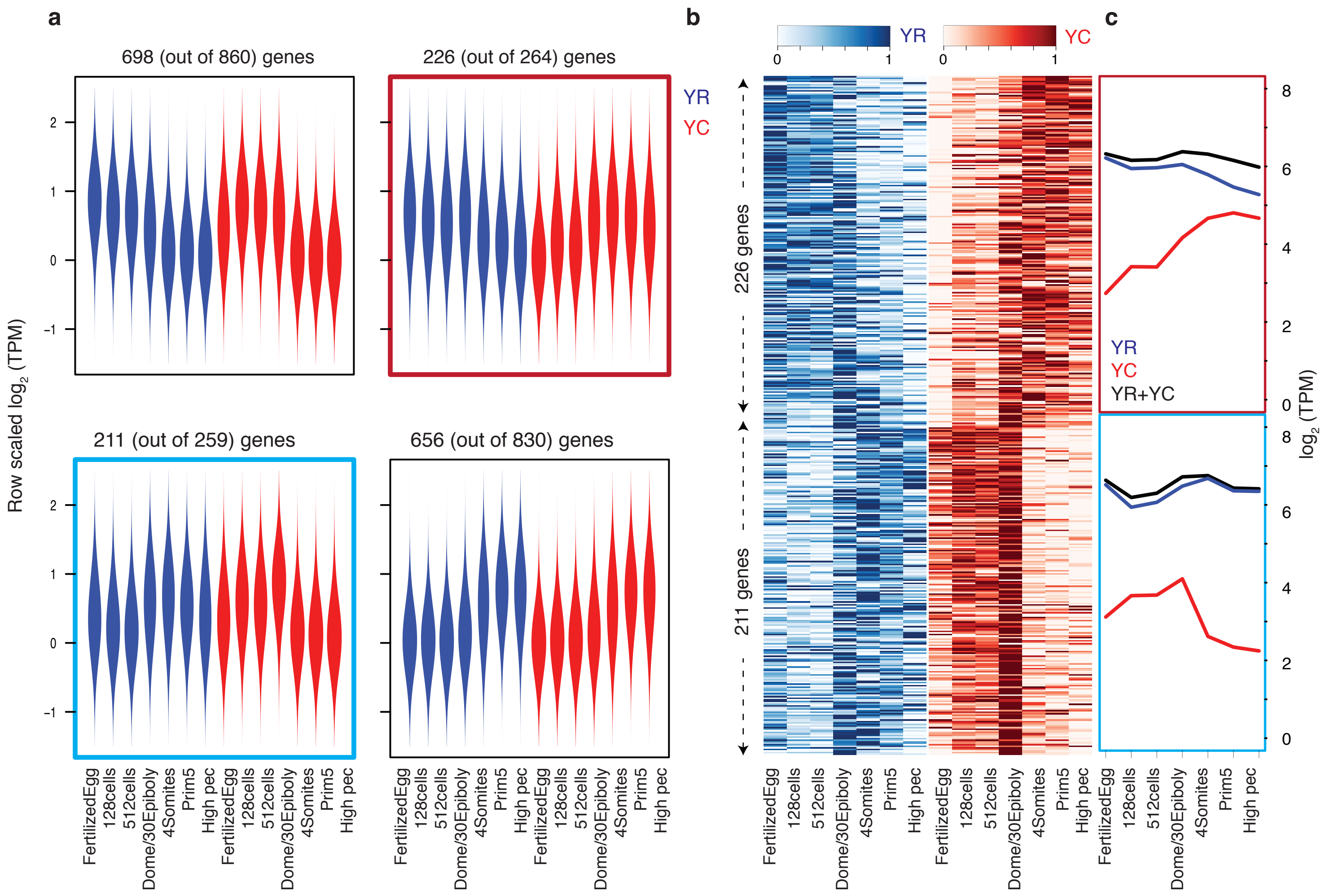

Supplementary Figure 3. Expression dynamics of YR-initiation and YC-initiation during zebrafish embryo development (**a**) Violin plot of expression profiles of YR and YC components of genes during maternal and zygotic stages in nAnTi CAGE-seq datasets. Dual initiation genes overlapping with respective classic CAGE clusters from the four corners of SOM matrix in Figure 3a are presented. Numbers without brackets represent numbers of genes detected in nAnTi CAGE from those detected by classic CAGE in the relevant clusters (gene number in brackets). Developmental stages are shown at the bottom. Y-axis indicates the scaled expression levels for YR and YC initiations. Blue and red colors indicate YR and YC components, respectively. (**b**) Heatmap rows show gene expression profiles with YR and YC-initiation of genes in nAnTi CAGE-seq data. Genes are presented from red and blue-framed violin plot clusters presented in a and indicated by the gene numbers. Expression values are scaled row-wise between 0 to 1, separately for YR and YC. Overlapping genes from nAnTi CAGE-seq are ranked as in classic CAGE data of Figure 3b. (**c**) Averaged expression level of total (black), YR-initiation (blue) and YC-initiation (red) across clustered group of genes in nAnTi CAGE-seq data presented in b.

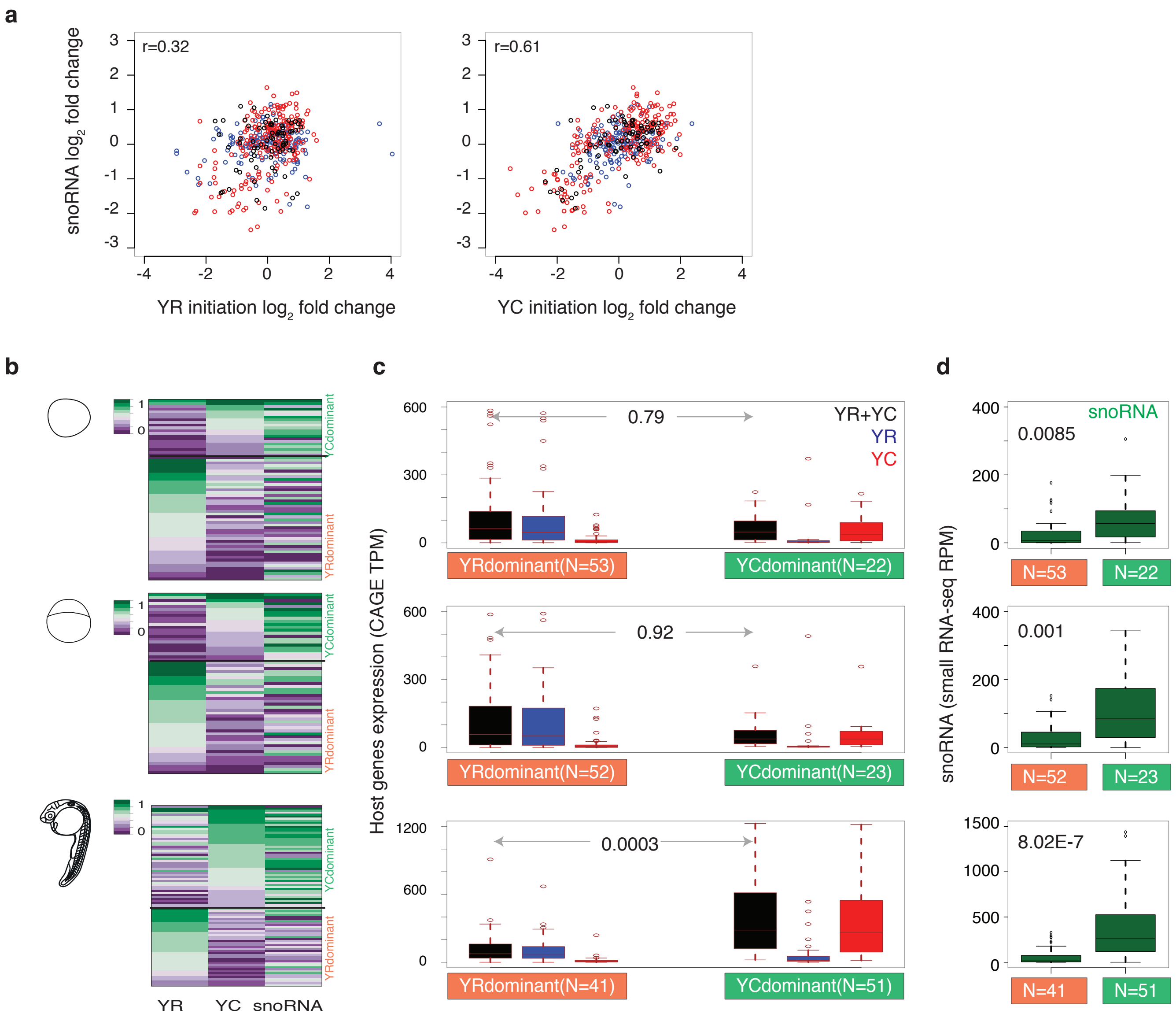

Supplementary Figure 4. Correlation of YR and YC components of snoRNA host genes with snoRNA expression levels. (**a**) Scatter plot of correlations between snoRNA log fold changes with YR initiation log fold changes (left), and with YC initiation log fold changes (middle) across 5 developmental stages of nAnTi CAGE data in 88 dual initiation snoRNA host genes Each point corresponds to one stage-gene pair of YR-dominant transcripts (blue), YC-dominant transcripts (red) and the rest (black). (**b**) Expression levels for YR, YC component of genes and that of their snoRNAs are visualized in heat map rows with ranking of YC and YR levels (high to low) respectively. YR and YC dominant genes are clustered and separated by a black line. (**c**) Expression levels of YR (blue) and YC (red) initiators, along with combined (black) expression levels of YR and YC. Host genes are divided into two groups (YR-dominant or YC-dominant) based on dominant expression of initiators. Y-axis indicate tags per million. (**d**) Expression levels of snoRNAs transcribed from YR and YC-dominant genes. Y-axis indicate reads per million.

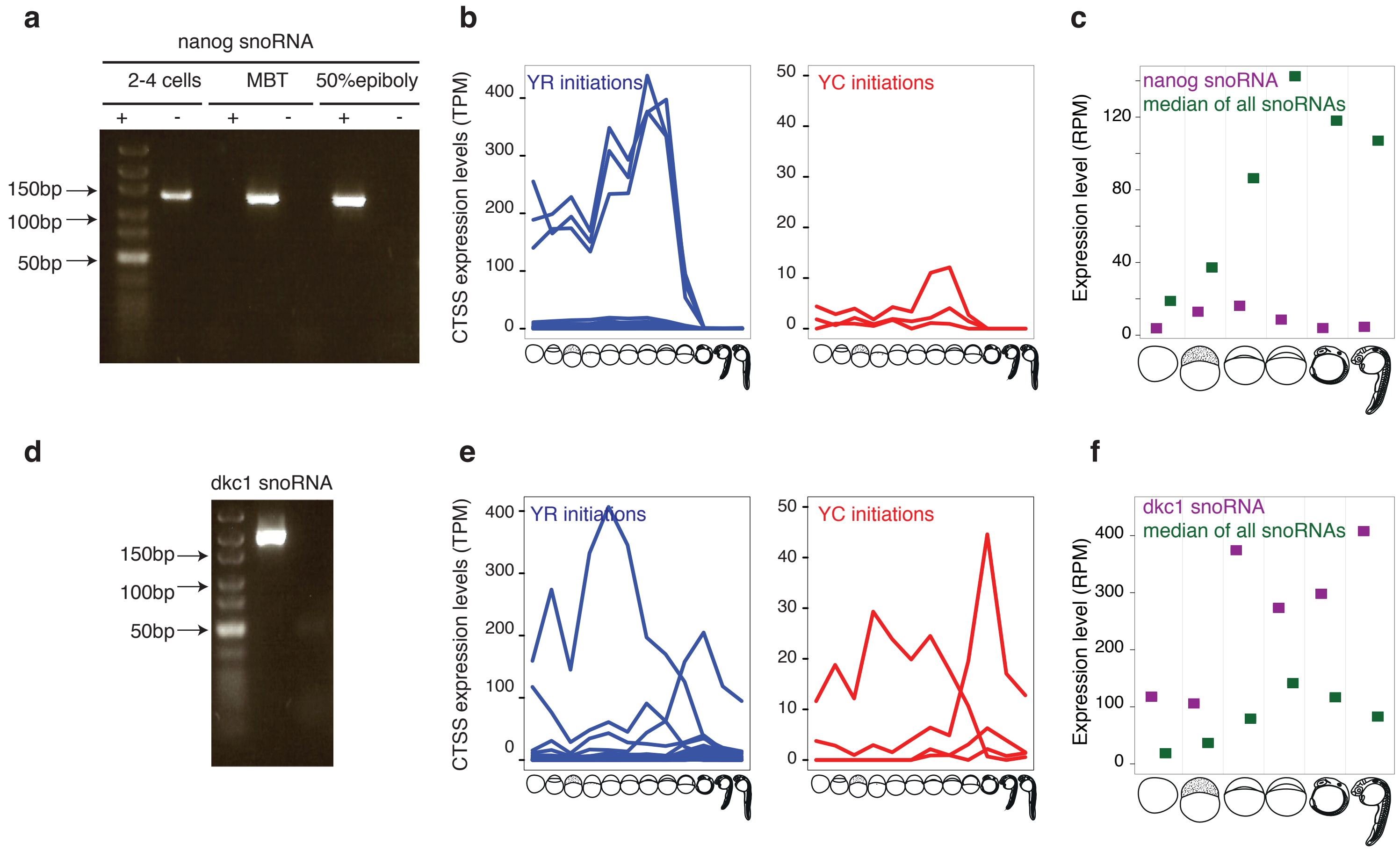

Supplementary Figure 5. Quantitation and dynamics of the expression of snoRNAs, and that of YC and YR-initiation of their host genes. (**a**) Validation of nanog snoRNA expression by RT-PCR in three developmental stages. Predicted size of PCR fragment is 131 bp. (**b**) Expression level and developmental dynamics of individual YR-initiation and YC-initiation in the nanog promoter region. X-axis indicate the developmental stages. (**c**) Expression (rpm) of a snoRNA encoded in the nanog host gene compared to mean expression of the rest of 93 snoRNA genes. (d) Validation of dkc1 snoRNA expression by RT-PCR in prim 5 stage. (**e**) Expression level and developmental dynamics of individual YR-initiation and YC-initiation in the dkc1 promoter region. X-axis indicate the developmental stages. (**f**) Expression (rpm) of a snoRNA encoded in the dkc1 gene as compared to mean expression of the rest of 93 snoRNA genes.

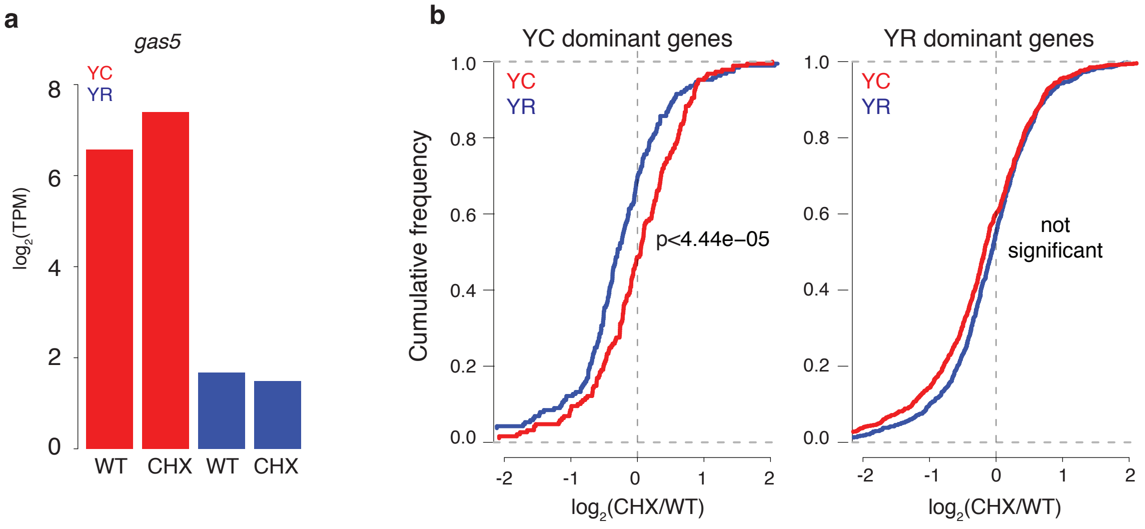

Supplementary Figure 6. Effect of translation inhibition on YR-initiation and YC-initiation products of dual-initiation promoters. (**a**) Bar chart of expression levels of YR-initiation and YC-initiation products of gas5 after cycloheximide treatment. Blue and red color indicates YR-initiation and YC-initiation respectively. (**b**) Cumulative frequency of YR-initiation and YC-initiation in YR-dominant (N=1771) and YC-dominant (N=241) genes. Abbreviations, CHX, cycloheximide treated, WT, wild type control embryos.

a

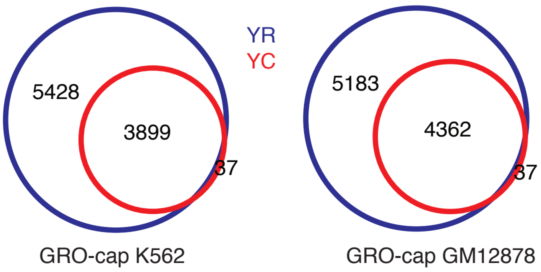

Supplementary Figure 7. Identification of dual initiation promoters in GRO-cap data. (**a**) Venn diagram with intersection of gene promoters with YR-initiation and YC-initiation in human K562 and GM12878 cell lines. Dual-initiation promoters are indicated in the overlap between detected YR-initiation and YC-initiation.
