# Supplementary material for: Dual-initiation promoters with intertwined canonical and TCT/TOP transcription start sites diversify transcript processing
Source: Nat Commun. 2020 Jan 10;11:168. doi: 10.1038/s41467-019-13687-0 (PMC6954239; doi:10.1038/s41467-019-13687-0)
Supplement: Supplementary file 2 — Description of Additional Supplementary Files [file 41467_2019_13687_MOESM2_ESM.pdf]

Description of Supplementary Data files:

Supplementary Data 1. Classification of genes into dual initiation promoters and single YR-only and YC-only initiator containing genes

Supplementary Data 2. Genes with YR and YC containing promoters in nAnTi CAGE datasets

Supplementary Data 3. Annotated novel snoRNAs and Ensembl annotated snoRNAs expressed during zebrafish embryogenesis

Supplementary Data 4. Gene Ontology (GO) analysis

Supplementary Data 5. Genes with negative coregulation of YR and YC during maternal to zygotic transition

Supplementary Data 6. Response of YR-initiation and YC-initiation after cycloheximide Treatment

Supplementary Data 7. Dual-initiation promoter genes in human HepG2 cell line and drosophila S2 cells from CAGE-seq

Supplementary Data 8. Dual-initiation promoter genes in human K562 and GM12878 cell lines from GRO-cap

Supplementary Data 9. tagging-CAGE CTSS table (normalised counts, tpm), Nepal et al. 2013, (doi: 10.1101/gr.153692.112)

Supplementary Data 10. Cycloheximide vs DMSO treated tagging-CAGE CTSS table (normalised counts, tpm).

Supplementary Data 11. snoRNA expression table (normalised counts, tpm), Lokati et al. 2017 (doi: 10.1261/rna.059642.116)

Supplementary Data 12. capped RNA-seq CTSS table (normalised counts, tpm).

Supplementary Data 13. nAnTi-CAGE CTSS table (normalised counts, tpm)
