# Supplementary material for: Dual-initiation promoters with intertwined canonical and TCT/TOP transcription start sites diversify transcript processing
Source: Nat Commun. 2020 Jan 10;11:168. doi: 10.1038/s41467-019-13687-0 (PMC6954239; doi:10.1038/s41467-019-13687-0)
Supplement: Supplementary file 16 — Reporting Summary [file 41467_2019_13687_MOESM16_ESM.pdf]

nature research

Corresponding author(s): Chirag Nepal and Ferenc Mueller

Last updated by author(s): Nov 14, 2019

Reporting Summary

Nature Research wishes to improve the reproducibility of the work that we publish. This form provides structure for consistency and transparency in reporting. For further information on Nature Research policies, see Authors & Referees and the Editorial Policy Checklist.

Statistics

For all statistical analyses, confirm that the following items are present in the figure legend, table legend, main text, or Methods section.

n/a	Confirmed	
☐	☒	The exact sample size (*n*) for each experimental group/condition, given as a discrete number and unit of measurement
☐	☒	A statement on whether measurements were taken from distinct samples or whether the same sample was measured repeatedly
☐	☒	The statistical test(s) used AND whether they are one- or two-sided<br>*Only common tests should be described solely by name; describe more complex techniques in the Methods section.*
☒	☐	A description of all covariates tested
☐	☒	A description of any assumptions or corrections, such as tests of normality and adjustment for multiple comparisons
☒	☐	A full description of the statistical parameters including central tendency (e.g. means) or other basic estimates (e.g. regression coefficient) AND variation (e.g. standard deviation) or associated estimates of uncertainty (e.g. confidence intervals)
☐	☒	For null hypothesis testing, the test statistic (e.g. *F*, *t*, *r*) with confidence intervals, effect sizes, degrees of freedom and *P* value noted<br>*Give P values as exact values whenever suitable.*
☒	☐	For Bayesian analysis, information on the choice of priors and Markov chain Monte Carlo settings
☐	☒	For hierarchical and complex designs, identification of the appropriate level for tests and full reporting of outcomes
☐	☒	Estimates of effect sizes (e.g. Cohen's *d*, Pearson's *r*), indicating how they were calculated

Our web collection on statistics for biologists contains articles on many of the points above.

Software and code

Policy information about availability of computer code

Data collection	Public data from FANTOM5, mod ENCODE and other individual papers were downloaded from the FTP site or through GEO accession as mentioned in the paper.
Data analysis	Data were analyzed by using publicly available softwares, which are cited in the paper. The version of software and syntax used are listed in the paper. All other custom code is available upon request

For manuscripts utilizing custom algorithms or software that are central to the research but not yet described in published literature, software must be made available to editors/reviewers. We strongly encourage code deposition in a community repository (e.g. GitHub). See the Nature Research guidelines for submitting code & software for further information.

Data

Policy information about availability of data

All manuscripts must include a data availability statement. This statement should provide the following information, where applicable:

- Accession codes, unique identifiers, or web links for publicly available datasets
- A list of figures that have associated raw data
- A description of any restrictions on data availability

Data availability statement has been included in the manuscript. Raw sequencing data are now publicly available at NCBI Sequence Read Archive (SRA). Processed data are provided as Supplementary Data files.

Field-specific reporting

Please select the one below that is the best fit for your research. If you are not sure, read the appropriate sections before making your selection.

☒ Life sciences ☐ Behavioural & social sciences ☐ Ecological, evolutionary & environmental sciences

For a reference copy of the document with all sections, see nature.com/documents/nr-reporting-summary-flat.pdf

Life sciences study design

All studies must disclose on these points even when the disclosure is negative.

Sample size	No statistical methods were used to predetermine sample size.
Data exclusions	No data were excluded from the analyses.
Replication	Reproducibility of the findings were replicated by generating an independent data set.
Randomization	Reproducibility of the findings were replicated by generating an independent data set.
Blinding	Blinding was not relevant for this study

Reporting for specific materials, systems and methods

We require information from authors about some types of materials, experimental systems and methods used in many studies. Here, indicate whether each material, system or method listed is relevant to your study. If you are not sure if a list item applies to your research, read the appropriate section before selecting a response.

Materials & experimental systems

n/a	Involved in the study	
☒	☐	Antibodies
☒	☐	Eukaryotic cell lines
☒	☐	Palaeontology
☐	☒	Animals and other organisms
☒	☐	Human research participants
☒	☐	Clinical data

Methods

n/a	Involved in the study	
☒	☐	ChIP-seq
☒	☐	Flow cytometry
☒	☐	MRI-based neuroimaging

Animals and other organisms

Policy information about studies involving animals; ARRIVE guidelines recommended for reporting animal research

Laboratory animals	Only early zebrafish embryos up to the free feeding stage (5 days post fertilization) were used in this study. Breeding and maintenance of adult zebrafish strains was carried out in a designated facility under Home Office project licenses 40/3681 and P51AB7F76 assigned to the University of Birmingham, UK.
Wild animals	The study did not involve wild animals
Field-collected samples	The study did not involve filed-collected samples
Ethics oversight	No ethical approval was required. Zebrafish embryos up to the free feeding stage are not considered as animals by law in the UK and are not subjected to animal experimentation regulations.

Note that full information on the approval of the study protocol must also be provided in the manuscript.
